# An extension of the best–worst method based on the spherical fuzzy sets for multi-criteria decision-making

**DOI:** 10.1007/s41066-024-00462-w

**Published:** 2024-03-30

**Authors:** Gholamreza Haseli, Reza Sheikh, Saeid Jafarzadeh Ghoushchi, Mostafa Hajiaghaei-Keshteli, Sarbast Moslem, Muhammet Deveci, Seifedine Kadry

**Affiliations:** 1 Tecnologico de Monterrey, School of Engineering and Sciences, Monterrey, Mexico; 2 School of Architecture Planning and Environmental Policy, University College Dublin, Belfield, Dublin, D04 V1W8 Ireland; 3 Faculty of Industrial Engineering and Management, Shahrood University of Technology, Shahrood, Iran; 4 Faculty of Industrial Engineering, Urmia University of Technology, Urmia, Iran; 5 Department of Industrial Engineering, Turkish Naval Academy, National Defence University, 34942 Tuzla, Istanbul, Turkey; 6 The Bartlett School of Sustainable Construction, University College London, 1-19 Torrington Place, London, WC1E 7HB UK; 7 Department of Electronical and Computer Engineering, Lebanese American University, Byblos, Lebanon; 8 Department of Applied Data Science, Noroff University College, Kristiansand, Norway; 9 Artificial Intelligence Research Center (AIRC), Ajman University, 346 Ajman, United Arab Emirates; 10 MEU Research Unit, Middle East University, Amman, 11831 Jordan

**Keywords:** Best–worst method, Spherical fuzzy sets, Multi-criteria decision-making, Fuzzy set, Consistency ratio

## Abstract

The ambiguous information in multi-criteria decision-making (MCDM) and the vagueness of decision-makers for qualitative judgments necessitate accurate tools to overcome uncertainties and generate reliable solutions. As one of the latest and most powerful MCDM methods for obtaining criteria weight, the best–worst method (BWM) has been developed. Compared to other MCDM methods, such as the analytic hierarchy process, the BWM requires fewer pairwise comparisons and produces more consistent results. Consequently, the main objective of this study is to develop an extension of BWM using spherical fuzzy sets (SFS) to address MCDM problems under uncertain conditions. Hesitancy, non-membership, and membership degrees are three-dimensional functions included in the SFS. The presence of three defined degrees allows decision-makers to express their judgments more accurately. An optimization model based on nonlinear constraints is used to determine optimal spherical fuzzy weight coefficients (SF-BWM). Additionally, a consistency ratio is proposed for the SF-BWM to assess the reliability of the proposed method in comparison to other versions of BWM. SF-BWM is examined using two numerical decision-making problems. The results show that the proposed method based on the SF-BWM provided the criteria weights with the same priority as the BWM and fuzzy BWM. However, there are differences in the criteria weight values based on the SF-BWM that indicate the accuracy and reliability of the obtained results. The main advantage of using SF-BWM is providing a better consistency ratio. Based on the comparative analysis, the consistency ratio obtained for SF-BWM is threefold better than the BWM and fuzzy BWM methods, which leads to more accurate results than BWM and fuzzy BWM.

## Introduction

The decision-making process is defined as selecting the best option among the existing options. Decision-makers are required to collect information from diverse sources, scrutinize the gathered data, and ultimately reach conclusive decisions to navigate the decision-making process (Hosseini et al. 2021; Rezazadeh et al. 2023). In intricate scenarios, decision-making relies on the effectiveness of multiple criteria. Consequently, the process of making decisions is referred to as multi-criteria decision-making (MCDM). MCDM methods empower decision-makers to make rational decisions considering several decision criteria (Haseli et al. 2023a). MCDM methods are used for two major decision-making tasks: weight determination of decision criteria and ranking alternatives. Various types of MCDM methods are developed in the literature to tackle weight determination and ranking alternatives, such as the Analytical Hierarchy Process (AHP) (Saaty 1987), Base-Criterion Method (BCM) (Haseli et al. 2020) the technique for order of preference by similarity to ideal solution (TOPSIS) (Hwang and Yoon 1981), VIseKriterijumska Optimizacija I Kompromisno Resenje (VIKOR) (Opricovic 1998), a mulTi-noRmalization mUlti-distance aSsessmenT (TRUST) (Torkayesh and Deveci 2021), LOgarithmic Percentage Change-driven Objective Weighting (LOPCOW) (Ecer and Pamucar 2022), and Halo Effect Convolutional Neural Networks (HECON) (Haseli et al. 2023a).

Due to the high popularity and applicability of MCDM methods in real-life problems, various new MCDM methods have been introduced in recent years. One of the most well-known of these methods is the best–worst method (BWM). BWM was developed by Rezaei (2015) based on a mathematical optimization model that employs a 1–9 scale to perform pairwise comparisons. Since then, BWM has been known as one of the most powerful methods to determine the criteria weight in addressing MCDM problems. The BWM overperforms traditional methods such as AHP and ANP as fewer pairwise comparisons are required in the BWM. Given these traits, BWM has captured the interest of scholars across various disciplines seeking to resolve MCDM issues with a focus on enhanced reliability and precision.

As decision problems become more intricate and parameters in real-life scenarios grow dynamic and uncertain, the dependability of outcomes produced by the conventional BWM is uncertain. This stems from the inability of the decision-makers to assign accurate values for pairwise comparison among criteria. In other words, this issue is due to data inaccuracy or subjectivity of quality in the evaluation of criteria by the decision-makers. Thus, to make optimal decisions in ambiguous and complex environments, the traditional BWM cannot generate reliable solutions for decision-making problems using crisp numbers (Rastpour et al. 2022; Vahidinia and Hasani 2023).

Given the intricacies of the contemporary world, arriving at logical decisions poses a formidable challenge within uncertain and ambiguous settings. In this regard, fuzzy sets have been considered an effective tool to empower decision-makers to deal with ambiguities and uncertainties (Mathew et al. 2020; Al-Zibaree and Konur 2023). For the first time, Zadeh (1975) introduced the concept of ordinary fuzzy sets. Since its development, fuzzy set theory has been a favorite topic for scientists in almost all branches of science, especially decision-making and decision sciences (Chen and Wang 1995, 2010; Chen et al. 2009; Horng et al. 2005; Chen and Jian 2017; Chen et al. 2019). The ordinary fuzzy sets successfully cover up ambiguity in decision-making issues but cannot control the hesitancy of ambiguous conditions (Gündoğdu 2022).

For this reason, advanced types of fuzzy sets are developed successfully to handle the data inaccuracy and uncertainty of decision problems (Chen 2022). One of these advanced types of fuzzy sets is called spherical fuzzy sets (SFS) (Kutlu Gundogdu and Kahraman 2019b). The SFS represents a recent advancement in fuzzy sets, introduced to address certain constraints observed in preceding fuzzy sets like Pythagorean fuzzy sets and Picture Fuzzy Sets. The main concept of SFS initiates from combining the scientifically approved aspects of Pythagorean fuzzy sets and Picture fuzzy sets (Donyatalab et al. 2022). The SFS provides a larger domain priority for decision-makers to determine their preferences than other fuzzy sets (Yildiz and Ozkan 2023). The membership function degrees of SFS can completely express individuals’ awareness and accurately in decision-making to illustrate the information of decisions with a scale that can flexibly regulate the range of information (Ali and Rashid 2019). The SFS successfully extended and has been used in solving decision-making problems in various fields (Akram and Ashraf 2023; Akram and Martino 2023; Zahid and Akram 2023; Bouraima et al. 2024; Hussain et al. 2024).

The BWM requires fewer pairwise comparisons to obtain the weight of criteria and provide a better consistency ratio for weight results (Rezaei 2015). In recent years, various extensions of BWM using several fuzzy sets were introduced to overcome the uncertainty and ambiguity in decision-making problems. Extensions of fuzzy BWM have been used successfully in different fields and applications (Sagnak et al. 2021; Zhou et al. 2022; Chen et al. 2023). Previous fuzzy extensions for the BWM method have been used in uncertain environments, but these fuzzy BWM extensions cannot consider the hesitancy degree of the decision-maker judgments. SFS enhances the effectiveness of the BWM technique by incorporating the degree of hesitation in decision-maker judgments. As far as our understanding goes, there is currently no expansion of SFS specifically designed for BWM.

This paper aims to use the SFS to extend the BWM to improve the decision-making environment under uncertain conditions. This method proposes a more comprehensive process to solve MCDM problems through its higher accuracy in evaluating pairwise comparisons of the decision-making process with better uncertainty measurements. In this method, decision-makers can express their hesitancy separately of the non-membership and membership degrees by satisfying the unit sphere statuses, which means the sum of the squared membership function must be at most equal to 1. Therefore, the SF-BWM can effectively represent the preferences and judgments in a more realistic form. To show the applicability and efficiency of the SF-BWM against previous extensions, two numerical problems have been investigated.

In this regard, the following contributions are considered for this research:

- Extending the BWM method under the SFS to consider the uncertainty of the decision-making.
- Using the proposed method, decision-makers can express their judgment hesitancy separately from the non-membership and membership degrees.
- The proposed method proposes a more comprehensive process to solve MCDM problems through its higher accuracy in evaluating pairwise comparisons of the decision-making process.
- The proposed method yields criteria weight results with a threefold improvement in consistency ratio.

The remainder of this paper is structured as follows. Section 2 presents the literature review on fuzzy sets and fuzzy BWM methods. In Sect. 3, the theoretical framework of the SF-BWM is introduced. In Sect. 4, two different problems are performed for performance evaluation, efficiency measurement, and validation of the SF-BWM. A comprehensive comparison between traditional BWM, fuzzy BWM, and SF-BWM methods are conducted in Sect. 5. Finally, the future directions and conclusions are provided in Sect. 6.

## Literature review

In recent years, various versions of fuzzy sets have been developed. Fuzzy sets are usually extended to increase the ability to control uncertainty and ambiguity in information. Due to the limitations of type 1 fuzzy sets, Zadeh (1975) introduced type 2 fuzzy sets a few years later. Considering the requirement to increase our ability to handle uncertainty in real-life information, interval type-2 fuzzy sets (Liang and Mendel 2000), Credal-based fuzzy number data clustering (Liu 2023), Enhanced fuzzy clustering for incomplete instances with evidence combination (Liu and Letchmunan 2024), and interval-valued fuzzy sets (Dubois 1980) were introduced to overcome the computational complexities of type 2 fuzzy sets. Later, intuitionistic fuzzy sets (Atanassov 1999) were proposed to consider human judgments for more accurate decision-making solutions. According to the principles of the intuitionistic fuzzy sets, the sum of membership and non-membership degrees must be equal to or less than 1, which sometimes violates this principle (Kutlu Gündoğdu 2020). In this regard, The membership function of a fuzzy set represents an extension of the classical set's indicator function. Zadeh (1975) proposed the membership function and determined a range of the interval covering [0,1], which operates on the domain of all possible values. The non-membership value equals 1—the membership value based on intuitionistic fuzzy sets. Pythagorean fuzzy sets are proposed to overcome the limitation of intuitionistic fuzzy sets (Yager 2013). The dependence of the hesitancy on the degree of non-membership and membership introduced the SFS. The degree of hesitancy in SFS is considered independent where each of the degrees is independently between 0 and 1.

By developing SFS by Kutlu Gundogdu and Kahraman (2019b), Ashraf et al. (2019) introduced weighted geometric aggregation operators and weighted averaging based on SFS to solve MCDM problems. Jin et al. (2019a) suggested a linguistic extension of SFS, which empowers decision-makers to handle defective and ambiguous information in decision-making problems. In addition, Jin et al. (2019b) introduced a new logarithmic operation approach to the SF-weighted geometric operators and SF-weighted average operators. Liu et al. (2020) introduced linguistic terms and then proposed the linguistic weight average operator for SFS. Lately, Ashraf et al. (2020) used intelligent SFS for emergency response decisions in diagnosing COVID-19.

Recently, several MCDM methods were extended based on the SFS. Kutlu Gundogdu and Kahraman (2019b), for the first time, introduced TOPSIS in a spherical fuzzy environment and then used the proposed methodology to handle a complex decision-making problem for the selection of 3D printers. Zeng et al. (2019) introduced a rough set-based SFS TOPSIS method for an illustrative problem. Kutlu Gündoğdu and Kahraman (2019a) developed a new extension of the VIKOR to handle the warehouse site selection problem under a spherical fuzzy environment. Later, Kutlu Gündoğdu and Kahraman (2019c) developed the WASPAS method based on SFS for industrial robot selection problem. In the same way, the SFS extension of the AHP method was developed for handling renewable energy decision problems (Gündoğdu and Kahraman 2020a). Liu et al. (2020) extended TODIM and MABAC methods using the linguistic SFS for evaluating the shared public bicycles problem in China. Gündoğdu and Kahraman (2020b) proposed a new spherical QFD for developing delta robot technology. Most recently, Shishavan et al. (2020) proposed the Jaccard, exponential, and square root cosine similarity scales for the SFS and used the new approach for green supplier selection and medical diagnosis problems. Haseli and Jafarzadeh Ghoushchi (2022) used the SFS to extend the BCM for solving the waste management problem. Also, Bonab et al. (2023) proposed a decision support model based on the integrated SFS with the Choquet integral approach to the assessment of the autonomous vehicles' logistics. Bouraima et al. (2023) used the Spherical fuzzy numbers to propose the group MCDM method to solve the sustainable regional transportation problem. Finally, Moslem (2024) introduced a new structure for decision-making with the combination of the Parsimonious concept under SFS to develop the AHP method.

After the development of the BWM by Rezaei (2015), this method was extended in different forms to be used in many fields and applications (Rahimi et al. 2020; Maghsoodi et al. 2020; Torkayesh and Simic 2022). Mou et al. (2016) introduced an intuitionistic fuzzy multiplicative preferential relations approach for the BWM. Later, Guo and Zhao (2017) proposed the fuzzy BWM and compared their results with classical BWM to show the lower consistency ratio of fuzzy BWM compared to classical BWM. Aboutorab et al. (2018) extended the BWM using the *Z*-number for supplier developments. The ZBWM method in addition to using fuzzy numbers, it takes to consider the degree of reliability of the decision-maker. The results show that the ZBWM method provides more accurate weights than the fuzzy BWM. In 2019, Ali and Rashid (2019) developed the hesitant fuzzy extension of the BWM. The case studies show that the hesitant fuzzy BWM lead to a higher consistency ratio of calculated weights than the BWM. Mi and Liao (2019) also proposed three different hesitant fuzzy BWM approaches to obtain the criteria weights considering the score-based model, decision-makers’ attitudes, and complete and non-missed pairwise values. Wu et al. (2019) introduced novel interval-type-2 fuzzy BWM and integrated it with the VIKOR method to select the green suppliers. Pishdar et al. (2019) used interval-type-2 fuzzy BWM for airport hop selection in different countries. Liu et al. (2020) introduced a fuzzy interval-valued Pythagorean hesitant extension of the BWM and applied it to select the 3PRLs (third-party reverse logistics) on a self-service mobile recycling machine. Vafadarnikjoo et al. (2020) proposed a novel neutrosophic enhanced BWM to consider decision makers' confidence. The proposed method is used in two supply chain management cases to evaluate the validity. Two real-world problems show that the neutrosophic-enhanced BWM led to a better consistency ratio than the BWM. Karimi et al. (2020) proposed a fully fuzzy BWM by the fuzzy triangular numbers and used this for evaluation of the maintenance in hospitals. Chen and Ming (2020) proposed a hybrid decision model using data envelopment analysis and the rough-fuzzy BWM method for smart product-service selection. Dong et al. (2021) proposed a novel fuzzy BWM using the concept of triangular fuzzy numbers. Also, Wan et al. (2021) introduced the generalized interval-valued trapezoidal fuzzy BWM based on the trapezoidal fuzzy numbers and provided three real-life examples to illustrate its application. Torkayesh et al. (2021) proposed a novel extension of BWM based on the concept of stratification to consider the impacts of future events through the weight determination environment. Stratified BWM approach was then applied to address two case studies for waste technology selection. Zhou et al. (2022) proposed an approach based on the extension of the BWM using the basic probability assignment in the Dempster–Shafer evidence theory. The proposed approach provides a promising way to ascertain the best basic probability assignment and worst basic probability assignment for conflict management. Also, Haseli et al. (2023b) proposed a novel group decision-making framework based on the BWM under fuzzy ZE-numbers to address the urban waste management problem by selecting sustainable resilient recycling partners. Finally, Moslem (2023) introduced a new structure for the BWM based on the Parsimonious concept.

### Research gap

As mentioned, in recent years most of the MCDM methods such as AHP, SWARA, VIKOR, TOPSIS, WASPAS, etc. have been developed using SFS to apply in the various scientific fields mentioned in the literature. Given the mentioned advantages of the SFS, it is necessary to develop a powerful MCDM method such as BWM using the SFS. In recent years, the BWM was developed using different fuzzy sets and fuzzy numbers, while so far researchers not have been developing the BWM using fuzzy SFS. This is an important research gap that the BWM has not been developing using the SFS.

The BWM is known as a popular MCDM method among researchers that has been able to get a lot of attention in a short time. Google Scholar statistics show that the BWM is widely applied to solve decision problems in different scientific fields as one of the most important MCDM methods. Also, various extensions of the BWM have been used for different applications. Therefore, due to the popularity, power, and wide uses of the BWM, as well as the advantages of the SFS, the development of the BWM using the SFS can be highly regarded and used to analyze the criteria weight in various scientific fields.

The utilization of SFS in the development of the BWM introduces the potential for enhancing the method's precision and applicability. By leveraging the unique characteristics of SFS, researchers can address the limitations associated with other fuzzy sets, providing a more comprehensive and nuanced approach to multi-criteria decision-making. As the BWM continues to gain traction as one of the most prominent MCDM methods, integrating SFS could contribute significantly to its versatility and effectiveness in analyzing criteria weights in diverse scientific fields. This innovative combination has the potential to advance decision-making methodologies, offering researchers and practitioners a more robust tool for tackling complex decision problems.

## Spherical fuzzy best–worst method

### Preliminaries of SFS

Kutlu Gundogdu and Kahraman (2019b) introduced the SFS which includes three degrees to express individuals’ awareness that can illustrate the information of decisions. Suppose *U* is the universe of discourse. Then, ${\mu }_{{\widetilde{A}}_{s}}\left(\mu \right)$*,* ${v}_{{\widetilde{A}}_{s}}\left(\mu \right)$ and ${\pi }_{{\widetilde{A}}_{s}}\left(\mu \right)$ are membership, non-membership, and hesitancy degrees, respectively. Therefor SFS ${\widetilde{A}}_{s}$ is defined as

1 $${\widetilde{A}}_{s}=\left\{\left(\mu , \left({\mu }_{{\widetilde{A}}_{s}}\left(u\right), {v}_{{\widetilde{A}}_{s}}\left(u\right),{\pi }_{{\widetilde{A}}_{s}}\left(u\right)\right)\right)\Big\vert u \epsilon U \right\}$$

$$\mathrm{Where }\,{\mu }_{{\widetilde{A}}_{s}}\left(\mu \right) :U\to \left[0, 1\right], {v}_{{\widetilde{A}}_{s}}\left(\mu \right) :U \to \left[\mathrm{0,1}\right], {\pi }_{{\widetilde{A}}_{s}}\left(\mu \right) :U \to [\mathrm{0,1}]$$

According to Eq. (2), the sum of the three degrees squares must have been equal or less to 1 (Kutlu Gundogdu and Kahraman 2019b).

2 $$0\le {\mu }_{{\widetilde{A}}_{s}}^{2}\left(u\right)+{v}_{{\widetilde{A}}_{s}}^{2}\left(u\right)+{\pi }_{{\widetilde{A}}_{s}}^{2}\left(u\right)\le 1 \quad \forall u\in U$$

Arithmetic operations of SFS such as addition, multiplication, multiplication with scalar, and power are defined as follows:

3 $${\widetilde{A}}_{s}\oplus {\widetilde{B}}_{s}=\left\{{\left({\mu }_{{\widetilde{A}}_{s}}^{2}+{\mu }_{{\widetilde{B}}_{s}}^{2}-{\mu }_{{\widetilde{A}}_{s}}^{2}{\mu }_{{\widetilde{B}}_{s}}^{2}\right)}^{1/2}, \,\, {v}_{{\widetilde{A}}_{s}}{v}_{{\widetilde{B}}_{s}}, {\left(\left(1-{\mu }_{{\widetilde{B}}_{s}}^{2}\right){\pi }_{{\widetilde{A}}_{s}}^{2}+\left(1-{\mu }_{{\widetilde{A}}_{s}}^{2}\right){\pi }_{{\widetilde{B}}_{s}}^{2}-{\pi }_{{\widetilde{A}}_{s}}^{2}{\pi }_{{\widetilde{B}}_{s}}^{2}\right)}^{1/2}\right\}$$

4 $${\widetilde{A}}_{s}\otimes {\widetilde{B}}_{s}=\left\{{\mu }_{{\widetilde{A}}_{s}}{\mu }_{{\widetilde{B}}_{s}}, {\left({v}_{{\widetilde{A}}_{s}}^{2}+{v}_{{\widetilde{B}}_{s}}^{2}-{v}_{{\widetilde{A}}_{s}}^{2}{v}_{{\widetilde{B}}_{s}}^{2}\right)}^{1/2},\, {\left(\left(1-{v}_{{\widetilde{B}}_{s}}^{2}\right){\pi }_{{\widetilde{A}}_{s}}^{2}+\left(1-{v}_{{\widetilde{A}}_{s}}^{2}\right){\pi }_{{\widetilde{B}}_{s}}^{2}-{\pi }_{{\widetilde{A}}_{s}}^{2}{\pi }_{{\widetilde{B}}_{s}}^{2}\right)}^{1/2}\right\}$$

5 $${\lambda \cdot \widetilde{A}}_{s}=\left\{{\left(1-{\left(1-{\mu }_{{\widetilde{A}}_{s}}^{2}\right)}^{\lambda }\right)}^{1/2},\,{v}_{{\widetilde{A}}_{s}}^{\lambda }, {\left({\left(1-{\mu }_{{\widetilde{A}}_{s}}^{2}\right)}^{\lambda }-{\left(1-{v}_{{\widetilde{A}}_{s}}^{2}-{\pi }_{{\widetilde{A}}_{s}}^{2}\right)}^{\lambda }\right)}^{1/2}\right\}$$

6 $${\widetilde{A}}_{s}^{\lambda }=\left\{{\mu }_{{\widetilde{A}}_{s}}^{\lambda },{\left(1-{\left(1-{v}_{{\widetilde{A}}_{s}}^{2}\right)}^{\lambda }\right)}^{1/2}, \,{\left({\left(1-{v}_{{\widetilde{A}}_{s}}^{2}\right)}^{\lambda }-{\left(1-{v}_{{\widetilde{A}}_{s}}^{2}-{\pi }_{{\widetilde{A}}_{s}}^{2}\right)}^{\lambda }\right) }^{1/2}\right\}$$

The addition and multiplication operators of SFS are used to find the criteria weights in the SF-BWM. For two SFS in the form of ${\widetilde{A}}_{s}$ and ${\widetilde{B}}_{s}$, following relations hold.

7 $${\widetilde{A}}_{s}\otimes {\widetilde{B}}_{s}={\widetilde{B}}_{s}\otimes {\widetilde{A}}_{s}$$

8 $${\widetilde{A}}_{s}\oplus {\widetilde{B}}_{s}={\widetilde{B}}_{s}\oplus {\widetilde{A}}_{s}$$

Gündoğdu and Kahraman (2021) proposed functions of the score and accuracy for SFS as follows:

9 $$\mathrm{Score }\left({\widetilde{A}}_{s}\right)={\left({\mu }_{{\widetilde{A}}_{s}}-{\pi }_{{\widetilde{A}}_{s}}\right)}^{2}-{\left({v}_{{\widetilde{A}}_{s}}-{\pi }_{{\widetilde{A}}_{s}}\right)}^{2}$$

10 $$\mathrm{Accuracy }\left({\widetilde{A}}_{s}\right)={\mu }_{{\widetilde{A}}_{s}}^{2}+{v}_{{\widetilde{A}}_{s}}^{2}+{\pi }_{{\widetilde{A}}_{s}}^{2}$$

Based on the equations of the (9) and (10), ${\widetilde{A}}_{s}<{\widetilde{B}}_{s}$ if only if

score $$\left({\widetilde{A}}_{s}\right)$$ < score $$\left({\widetilde{B}}_{s}\right)$$

or

score $\left({\widetilde{A}}_{s}\right)$ = score $\left({\widetilde{B}}_{s}\right)$ and accuracy $\left({\widetilde{A}}_{s}\right)$ < accuracy $\left({\widetilde{B}}_{s}\right)$

Sometimes, the values of these functions are inappropriate. For example, the score function value may be negative or zero. The spherical fuzzy preference score function (0.5, 0.5, 0.5) is zero. In addition, the score function of spherical fuzzy preference (0.3, 0.7, 0.3) is negative “0.16”. Moreover, two different spherical fuzzy preferences may have a similar score function. Furthermore, the accuracy function may provide a similar value for two spherical fuzzy preferences similar to the score function. Considering two spherical fuzzy preferences of (0.7, 0.3, 0.3) and (0.3, 0.7, 0.3), the result of the accuracy function is a similar value “0.42” (Sharaf 2021). As a result, the prioritization function is defined for spherical fuzzy preferences based on Eq. (11).

11 $$F\left({\widetilde{A}}_{s}\right)=\left({\mu }_{{\widetilde{A}}_{s}}\right)*\left(1-{v}_{{\widetilde{A}}_{s}}\right)*\left(1-{\pi }_{{\widetilde{A}}_{s}}\right)$$

### Spherical fuzzy BWM

In the MCDM methods, decision-makers are performed pairwise comparisons between all criteria to find the criteria weights. For example, in the AHP method, to obtain the optimal weight of n criteria should be executed $n\left(n-1\right)/2$ pairwise comparisons are required between criteria (Haseli et al. 2020). Rezaei (2015) introduced the BWM, which shows that decision-makers can obtain criteria weights by performing the $2n-3$ pairwise comparisons. The BWM is vector-based. In this method, pairwise comparisons are divided as reference and secondary. The results show that initial pairwise comparisons are sufficient to find the optimal criteria weight (Cheraghalipour et al. 2018; Haseli et al. 2021). In this paper, the BWM is extended by spherical fuzzy. Reference pairwise comparisons are performed in the new method using the SFS functions. To handle decision-making problems under conditions of ambiguity and uncertainty by using the SF-BWM, the following steps are followed:

*Step 1*. Determine the set of decision-making criteria. For example, some of criteria $\left\{{C}_{1},{C}_{2},{C}_{3},\cdots ,{C}_{n}\right\}$ affects decision-making problem.

In this regard, the effective criteria for each decision-making problem should be determined at the first stage of the solving process.

*Step 2*. Define the best and worst criteria.

In this step, the decision-makers should define the most important criterion as the best one. Also, they should define the weakly importance criterion as the worst criterion.

*Step 3*. Evaluate the spherical fuzzy preference of the best criterion over all the other criteria using the spherical fuzzy numbers based on Table 1. As shown in Table 1, the decision makers' preferences are considered three values for hesitancy, non-membership, and membership degrees. The spherical fuzzy preference of (0.9, 0.1, 0.1) indicates extreme preference. Also, equal preference is indicated by (0.5, 0.5, 0.5).

**Table 1:** The preference scales of spherical fuzzy (Kutlu Gündoğdu and Kahraman 2019a)

Linguistic terms	$\left(\mu , v, \pi \right)$
Extremely not preference	(0.1, 0.9, 0.1)
Very strongly not preference	(0.2, 0.8, 0.2)
Strongly not preference	(0.3, 0.7, 0.3)
Moderately not preference	(0.4, 0.6, 0.4)
Equally preference	(0.5, 0.5, 0.5)
Moderately preference	(0.6, 0.4, 0.4)
Strongly preference	(0.7, 0.3, 0.3)
Very strongly preference	(0.8, 0.2, 0.2)
Extremely preference	(0.9, 0.1, 0.1)

The resulting spherical fuzzy preference of the best-to-other criteria will be as follows:

12 $${\widetilde{A}}_{sB}=\left(\left({\mu }_{B1},{ v}_{B1},{ \pi }_{B1}\right),\left({\mu }_{B2},{ v}_{B2},{ \pi }_{B2}\right),\cdots ,\left({\mu }_{Bn},{ v}_{Bn},{ \pi }_{Bn}\right)\right)$$

According to problem (12), the relative importance values of the best criterion to all criteria are determined based on the SFS variables.

*Step 4*. Evaluate the spherical fuzzy preference of the vector using the spherical fuzzy linguistic variables based on Table 1. The resulting spherical fuzzy preference of step 4 will be as follows:

13 $${\widetilde{A}}_{sw}=\left(\left({\mu }_{1w},{ v}_{1w},{ \pi }_{1w}\right),\left({\mu }_{2w},{ v}_{2w},{ \pi }_{2w}\right),\cdots ,\left({\mu }_{nw},{ v}_{nw},{ \pi }_{nw}\right)\right)$$

According to problem (13), the relative importance values of each criterion to the worst criterion are determined based on the SFS variables.

*Step 5*. Find the spherical fuzzy criteria weight. In the BWM, the criteria weight for each of ${w}_{j}/{w}_{w}$ and ${w}_{B}/{w}_{j}$, it should have ${w}_{j}/{w}_{w}={a}_{Bj}$ and ${w}_{B}/{w}_{j}={a}_{jw}$. The maximum absolute gaps $\left|\frac{{w}_{j}}{{w}_{w}}-{a}_{jw}\right|$ and $\left|\frac{{w}_{B}}{{w}_{j}}-{a}_{Bj}\right|$ for criteria can be found using the nonlinear mathematical program (Rezaei 2015). According to the sum of the conditions, the criteria weights can be found by deriving the following problem:

$${\text{Minmax}}\left\{\left|\frac{{w}_{B}}{{w}_{j}}-{a}_{Bj}\right|, \left|\frac{{w}_{j}}{{w}_{w}}-{a}_{jw}\right|\right\}$$

$$\mathrm{Such\,that}$$

$$\sum_{j}{w}_{j}=1$$

14 $${w}_{j}\ge 0\quad \mathrm{ for\, all }\,j$$

Problem (14) can be rewritten as the nonlinear problem as follows:

$$\mathrm{Min \xi }$$

$$\mathrm{ Such\,that}$$

15 $$\left\{\begin{array}{l}\vert\frac{{w}_{B}}{{w}_{j}}-{a}_{Bj}\vert \le \xi \\ \vert\frac{{w}_{j}}{{w}_{w}}-{a}_{jw}\vert \le \xi \\ \sum\limits_{j=1}^{n}w{\text{j}}=1 \\ {w}_{j}\ge 0 \quad \mathrm{for\,all}\,j\end{array}\right.$$

The SF-BWM used the spherical fuzzy numbers $\left(\mu , v, \pi \right)$ instead of crisp numbers for ${w}_{B}$, ${w}_{j}$ and ${w}_{w}$. The sum of the spherical fuzzy weights should be equal to one. This limitation can be determined in the SF-BWM based on Eq. (11). Also, the sum of the non-membership and membership degrees must be large or equal to 0 and less or equal to 1. Therefore, problem (15) can be converted to the following problem by spherical fuzzy numbers.

$$\mathrm{Min }\,\left({\mu }_{k}, {v}_{k}, {\pi }_{k}\right)$$

$$\mathrm{Such\,that}$$

16 $$\left\{\begin{array}{l}\left|\frac{\left({\mu }_{B}^{w}, {v}_{B}^{w}, {\pi }_{B}^{w}\right)}{\left({\mu }_{j}^{w}, {v}_{j}^{w}, {\pi }_{j}^{w}\right)}-\left({\mu }_{Bj}, {v}_{Bj}, {\pi }_{Bj}\right)\right|\le \left({\mu }_{k}, {v}_{k}, {\pi }_{k}\right) \\ \left|\frac{\left({\mu }_{j}^{w}, {v}_{j}^{w}, {\pi }_{j}^{w}\right)}{\left({\mu }_{w}^{w}, {v}_{w}^{w}, {\pi }_{w}^{w}\right)}-\left({\mu }_{jw}, {v}_{jw}, {\pi }_{jw}\right)\right|\le \left({\mu }_{k}, {v}_{k}, {\pi }_{k}\right)\\ \sum\limits_{j=1}^{n}\left(\left({\mu }_{j}\right)*\left(1-{v}_{j}\right)*\left(1-{\pi }_{j}\right)\right)=1 \\ 0\le {\mu }_{j}+{v}_{j}\le 1\, \mathrm{for\,all}\,j \\ 0\le {\mu }_{j}^{2}+{v}_{j}^{2}+{\pi }_{j}^{2}\le 1\, \mathrm{for\,all}\, j \end{array}\right.$$

According to the preliminaries of SFS for addition and multiplication operations, problem (16) can be transferred to problem (17). In this regard, the *Z* is defined instead of the ξ in Eq. (15).

$$\mathrm{Min }Z=\left({\mu }_{k}\right)*\left(1-{v}_{k}\right)*\left(1-{\pi }_{k}\right)$$

$$\mathrm{Such\,that}$$

17 $$\left\{\begin{array}{l}\left({\mu }_{B}^{w}, {v}_{B}^{w}, {\pi }_{B}^{w}\right)\le \left({\mu }_{k}, {v}_{k}, {\pi }_{k}\right)\left({\mu }_{j}^{w}, {v}_{j}^{w}, {\pi }_{j}^{w}\right)+\left({\mu }_{Bj}, {v}_{Bj}, {\pi }_{Bj}\right)\left({\mu }_{j}^{w}, {v}_{j}^{w}, {\pi }_{j}^{w}\right) \\ \left({\mu }_{B}^{w}, {v}_{B}^{w}, {\pi }_{B}^{w}\right)\ge -\left(\left({\mu }_{k}, {v}_{k}, {\pi }_{k}\right)\left({\mu }_{j}^{w}, {v}_{j}^{w}, {\pi }_{j}^{w}\right)+\left({\mu }_{Bj}, {v}_{Bj}, {\pi }_{Bj}\right)\left({\mu }_{j}^{w}, {v}_{j}^{w}, {\pi }_{j}^{w}\right)\right)\\ \left({\mu }_{j}^{w}, {v}_{j}^{w}, {\pi }_{j}^{w}\right)\le \left({\mu }_{k}, {v}_{k}, {\pi }_{k}\right)\left({\mu }_{w}^{w}, {v}_{w}^{w}, {\pi }_{w}^{w}\right)+\left({\mu }_{jw}, {v}_{jw}, {\pi }_{jw}\right)\left({\mu }_{w}^{w}, {v}_{w}^{w}, {\pi }_{w}^{w}\right) \\ \left({\mu }_{j}^{w}, {v}_{j}^{w}, {\pi }_{j}^{w}\right)\ge -\left(\left({\mu }_{k}, {v}_{k}, {\pi }_{k}\right)\left({\mu }_{w}^{w}, {v}_{w}^{w}, {\pi }_{w}^{w}\right)+\left({\mu }_{jw}, {v}_{jw}, {\pi }_{jw}\right)\left({\mu }_{w}^{w}, {v}_{w}^{w}, {\pi }_{w}^{w}\right)\right)\\ \sum\limits_{j=1}^{n}\left(\left({\mu }_{j}\right)*\left(1-{v}_{j}\right)*\left(1-{\pi }_{j}\right)\right)=1 \\ 0\le {\mu }_{j}+{v}_{j}\le 1 \,\mathrm{for\,all} \,j \\ 0\le {\mu }_{j}^{2}+{v}_{j}^{2}+{\pi }_{j}^{2}\le 1 \,\mathrm{for\,all} \,j \end{array}\right.$$

By applying the SFS addition and multiplication operations on the problem (17), the following result is obtained:

$$Z=\left({\mu }_{k}\right)*\left(1-{v}_{k}\right)*\left(1-{\pi }_{k}\right)$$

$$\mathrm{ Such\,that }$$

$$\left({\mu }_{B}^{w}, {v}_{B}^{w}, {\pi }_{B}^{w}\right)\le \left({{\left({\mu }_{k}{\mu }_{j}\right)}^{2}+{\left({\mu }_{Bj}{\mu }_{j}\right)}^{2}-\left({\left({\mu }_{k}{\mu }_{j}\right)}^{2}*{\left({\mu }_{Bj}{\mu }_{j}\right)}^{2}\right)}^\frac{1}{2}, {\left({v}_{k}^{2}+{v}_{j}^{2}-{v}_{k}^{2}{v}_{j}^{2}\right)}^\frac{1}{2}*{\left({v}_{Bj}^{2}+{v}_{j}^{2}-{v}_{Bj}^{2}{v}_{j}^{2}\right)}^\frac{1}{2}, {\left(\left(\left(1-{\left({\mu }_{Bj}{\mu }_{j}\right)}^{2}\right)\left(\left(1-{v}_{j}^{2}\right){\pi }_{k}^{2}+\left(1-{v}_{k}^{2}\right){\pi }_{j}^{2}-{\pi }_{k}^{2}{\pi }_{j}^{2}\right)\right)+\left(\left(1-{\left({\mu }_{k}{\mu }_{j}\right)}^{2}\right)\left(\left(1-{v}_{j}^{2}\right){\pi }_{Bj}^{2}+\left(1-{v}_{Bj}^{2}\right){\pi }_{j}^{2}-{\pi }_{Bj}^{2}{\pi }_{j}^{2}\right)\right)-\left(\left(\left(1-{v}_{j}^{2}\right){\pi }_{k}^{2}+\left(1-{v}_{k}^{2}\right){\pi }_{j}^{2}-{\pi }_{k}^{2}{\pi }_{j}^{2}\right)\left(\left(1-{v}_{j}^{2}\right){\pi }_{Bj}^{2}+\left(1-{v}_{Bj}^{2}\right){\pi }_{j}^{2}-{\pi }_{Bj}^{2}{\pi }_{j}^{2}\right)\right)\right)}^{1/2}\right)$$

$$\left({\mu }_{B}^{w}, {v}_{B}^{w}, {\pi }_{B}^{w}\right)\ge -\left({{\left({\mu }_{k}{\mu }_{j}\right)}^{2}+{\left({\mu }_{Bj}{\mu }_{j}\right)}^{2}-\left({\left({\mu }_{k}{\mu }_{j}\right)}^{2}*{\left({\mu }_{Bj}{\mu }_{j}\right)}^{2}\right)}^\frac{1}{2}, {\left({v}_{k}^{2}+{v}_{j}^{2}-{v}_{k}^{2}{v}_{j}^{2}\right)}^\frac{1}{2}*{\left({v}_{Bj}^{2}+{v}_{j}^{2}-{v}_{Bj}^{2}{v}_{j}^{2}\right)}^\frac{1}{2}, {\left(\left(\left(1-{\left({\mu }_{Bj}{\mu }_{j}\right)}^{2}\right)\left(\left(1-{v}_{j}^{2}\right){\pi }_{k}^{2}+\left(1-{v}_{k}^{2}\right){\pi }_{j}^{2}-{\pi }_{k}^{2}{\pi }_{j}^{2}\right)\right)+\left(\left(1-{\left({\mu }_{k}{\mu }_{j}\right)}^{2}\right)\left(\left(1-{v}_{j}^{2}\right){\pi }_{Bj}^{2}+\left(1-{v}_{Bj}^{2}\right){\pi }_{j}^{2}-{\pi }_{Bj}^{2}{\pi }_{j}^{2}\right)\right)-\left(\left(\left(1-{v}_{j}^{2}\right){\pi }_{k}^{2}+\left(1-{v}_{k}^{2}\right){\pi }_{j}^{2}-{\pi }_{k}^{2}{\pi }_{j}^{2}\right)\left(\left(1-{v}_{j}^{2}\right){\pi }_{Bj}^{2}+\left(1-{v}_{Bj}^{2}\right){\pi }_{j}^{2}-{\pi }_{Bj}^{2}{\pi }_{j}^{2}\right)\right)\right)}^{1/2}\right)$$

$$\left({\mu }_{j}^{w}, {v}_{j}^{w}, {\pi }_{j}^{w}\right)\le \left({{\left({\mu }_{k}{\mu }_{w}\right)}^{2}+{\left({\mu }_{jw}{\mu }_{w}\right)}^{2}-\left({\left({\mu }_{k}{\mu }_{w}\right)}^{2}*{\left({\mu }_{jw}{\mu }_{w}\right)}^{2}\right)}^\frac{1}{2}, {\left({v}_{k}^{2}+{v}_{w}^{2}-{v}_{k}^{2}{v}_{w}^{2}\right)}^\frac{1}{2}*{\left({v}_{jw}^{2}+{v}_{w}^{2}-{v}_{jw}^{2}{v}_{w}^{2}\right)}^\frac{1}{2}, {\left(\left(\left(1-{\left({\mu }_{jw}{\mu }_{w}\right)}^{2}\right)\left(\left(1-{v}_{w}^{2}\right){\pi }_{k}^{2}+\left(1-{v}_{k}^{2}\right){\pi }_{w}^{2}-{\pi }_{k}^{2}{\pi }_{w}^{2}\right)\right)+\left(\left(1-{\left({\mu }_{k}{\mu }_{w}\right)}^{2}\right)\left(\left(1-{v}_{w}^{2}\right){\pi }_{jw}^{2}+\left(1-{v}_{jw}^{2}\right){\pi }_{w}^{2}-{\pi }_{jw}^{2}{\pi }_{w}^{2}\right)\right)-\left(\left(\left(1-{v}_{w}^{2}\right){\pi }_{k}^{2}+\left(1-{v}_{k}^{2}\right){\pi }_{w}^{2}-{\pi }_{k}^{2}{\pi }_{w}^{2}\right)\left(\left(1-{v}_{w}^{2}\right){\pi }_{jw}^{2}+\left(1-{v}_{jw}^{2}\right){\pi }_{w}^{2}-{\pi }_{jw}^{2}{\pi }_{w}^{2}\right)\right)\right)}^{1/2}\right)$$

$$\left({\mu }_{j}^{w}, {v}_{j}^{w}, {\pi }_{j}^{w}\right)\ge -\left({{\left({\mu }_{k}{\mu }_{w}\right)}^{2}+{\left({\mu }_{jw}{\mu }_{w}\right)}^{2}-\left({\left({\mu }_{k}{\mu }_{w}\right)}^{2}*{\left({\mu }_{jw}{\mu }_{w}\right)}^{2}\right)}^\frac{1}{2}, {\left({v}_{k}^{2}+{v}_{w}^{2}-{v}_{k}^{2}{v}_{w}^{2}\right)}^\frac{1}{2}*{\left({v}_{jw}^{2}+{v}_{w}^{2}-{v}_{jw}^{2}{v}_{w}^{2}\right)}^\frac{1}{2}, {\left(\left(\left(1-{\left({\mu }_{jw}{\mu }_{w}\right)}^{2}\right)\left(\left(1-{v}_{w}^{2}\right){\pi }_{k}^{2}+\left(1-{v}_{k}^{2}\right){\pi }_{w}^{2}-{\pi }_{k}^{2}{\pi }_{w}^{2}\right)\right)+\left(\left(1-{\left({\mu }_{k}{\mu }_{w}\right)}^{2}\right)\left(\left(1-{v}_{w}^{2}\right){\pi }_{jw}^{2}+\left(1-{v}_{jw}^{2}\right){\pi }_{w}^{2}-{\pi }_{jw}^{2}{\pi }_{w}^{2}\right)\right)-\left(\left(\left(1-{v}_{w}^{2}\right){\pi }_{k}^{2}+\left(1-{v}_{k}^{2}\right){\pi }_{w}^{2}-{\pi }_{k}^{2}{\pi }_{w}^{2}\right)\left(\left(1-{v}_{w}^{2}\right){\pi }_{jw}^{2}+\left(1-{v}_{jw}^{2}\right){\pi }_{w}^{2}-{\pi }_{jw}^{2}{\pi }_{w}^{2}\right)\right)\right)}^\frac{1}{2}\right)$$

$$\sum\limits_{j=1}^{n}\left(\left({\mu }_{j}\right)*\left(1-{v}_{j}\right)*\left(1-{\pi }_{j}\right)\right)=1$$

$$0\le {\mu }_{j}+{v}_{j}\le 1\,\mathrm{ for\,all}\, j$$

18 $$0\le {\mu }_{j}^{2}+{v}_{j}^{2}+{\pi }_{j}^{2}\le 1\mathrm{ for\,all \,}j$$

By handling problem (18), the spherical fuzzy weights of criteria can be obtained.

### Consistency ratio of SF-BWM

Rezaei (2015) introduced a consistency index to assess preference information in the context of crisp values. However, because the existing consistency index for conventional BWM is not appropriate for spherical fuzzy values, we have developed a new consistency index specifically for BWM when dealing with spherical fuzzy values.

Pairwise comparisons are fully consistent when ${\widetilde{a}}_{Bj}\times {\widetilde{a}}_{jw}={\widetilde{a}}_{Bw}$. In case of ${\widetilde{a}}_{Bj}\times {\widetilde{a}}_{jw}\ne {\widetilde{a}}_{Bw}$, an inconsistency would occur in the spherical fuzzy pairwise comparisons. Therefore, the spherical fuzzy value of *Z* mentioned in Eq. (17) should be decreased from ${\widetilde{a}}_{Bj}$ and ${\widetilde{a}}_{jw}$ and then to be added to ${\widetilde{a}}_{Bw}$, or equivalently based on Eq. (19).

19 $$\left({\widetilde{a}}_{Bj}-Z\right)\times \left({\widetilde{a}}_{jw}-Z\right)=\left({\widetilde{a}}_{Bw}+Z\right)$$

If ${\widetilde{a}}_{Bj}$ and ${\widetilde{a}}_{jw}$ are equal to ${\widetilde{a}}_{Bw}$, the inconsistency ratio will reach the highest value. Thus, Eq. (19) can be re-written as Eq. (20).

20 $$\left({\widetilde{a}}_{Bw}-Z\right)\times \left({\widetilde{a}}_{Bw}-Z\right)=\left({\widetilde{a}}_{Bw}+Z\right)$$

Equation (20) can be solved in the following form.

21 $${Z}^{2}-\left(1+2{\widetilde{a}}_{Bw}\right)Z+\left({{\widetilde{a}}_{Bw}}^{2}-{\widetilde{a}}_{Bw}\right)=0$$

Based on Table 1, the maximum possible value of spherical fuzzy preferences ${a}_{Bw}$ is (0.9, 0.1, 0.1), which decision-makers can assign. Also, the spherical fuzzy value of (0.5, 0.5, 0.5) is used by decision-makers to indicate the “Equal preference”. The spherical fuzzy values (0.1, 0.9, 0.1) to (0.4, 0.6, 0.4) do not apply to the SF-BWM. Since, according to the framework of the BWM, only positive preferences can be used as decision-maker’s preferences. The spherical fuzzy values (0.1, 0.9, 0.1) to (0.4, 0.6, 0.4) indicate the inverse of positive preferences. In this regard, although the inverse of positive preferences can be used in some MCDM methods such as AHP, it cannot be used in SF-BWM due to the framework of BWM which only accepts positive preferences. Therefore, the consistency index is according to Table 2 for the different spherical fuzzy preferences of the SF-BWM.

**Table 2:** Consistency index of spherical fuzzy preferences

Linguistic terms	Equally preference	Moderately preference	Strongly preference	Very strongly preference	Extremely preference
${\widetilde{a}}_{Bw}$	(0.5, 0.5, 0.5)	(0.6, 0.4, 0.4)	(0.7, 0.3, 0.3)	(0.8, 0.2, 0.2)	(0.9, 0.1, 0.1)
CI	3.00	3.80	5.29	6.69	8.04

By solving Eq. (18) for different spherical fuzzy preferences, values of *Z* can be obtained. The value of *Z* shows the error ranges in the obtained results. Based on the provided values for the CI in Table 2, the value range for the *Z* is between 0 to 8.04. These values can be used to calculate the consistency ratio. The consistency ratio is determined by replacing the consistency index and obtained *Z* values in Eq. (22). For this purpose, consistency index value can be found based on ${\widetilde{a}}_{Bw}$ in Table 2. According to Eq. (22), if the value of *Z* is equal to zero, then the spherical fuzzy pairwise comparisons will be fully consistent. Furthermore, the maximum value for *Z* is 8.04, signifying a consistency ratio of 1. This result indicates the highest level of inconsistency in the obtained weights.

22 $$\mathrm{Consistency\,ratio}= \frac{{\text{Z}}}{\mathrm{Consistency\,Index}}$$

## Illustrative problems

In this section, two different numerical problems are presented to show the applicability and efficiency of the SF-BWM for real-life MCDM problems compared to traditional BWM and fuzzy BWM. LINGO software is used to model the SF-BWM to obtain the spherical fuzzy weights of criteria.

### Problem I: selection of transportation mode

Rezaei (2015) presented a simple numerical problem for the selection of transportation modes to show the applicability of the traditional BWM. In the same way, the transportation mode selection problem is adopted to be solved by the SF-BWM under uncertain conditions. SF-BWM empowers us to consider the intangibility and ambiguity of decision-makers in pairwise comparisons. According to the first step mentioned in Sect. 3.2, the affective criteria should be determined. All effective criteria for selecting the best transportation mode are determined by the decision-maker. There are three decision criteria for selecting the transportation mode of products to a market (step 1). Load flexibility (C_1_), accessibility (C_2_), and cost (C_3_) are four criteria of the problem given by (Rezaei 2015). According to step 2 mentioned in Sect. 3.2, the decision-maker chooses the best and worst criteria based on the mentioned points regarding the most and weakly important criteria. In this regard, the cost (C_3_) criterion is chosen as the best criterion and also load flexibility (C_1_) criterion is chosen as the worst criterion. After determining the effective criteria in step 1 and choosing the best and worst criteria in step 2, the pairwise comparisons are performed based on the mentioned ways in steps 3 and 4 of Sect. 3.2. In step 3, performs pairwise comparisons for the best criterion against all other criteria. In step 4, similar comparisons were conducted for all criteria against the worst criterion. All pairwise comparisons of steps 3 and 4 are performed using the spherical fuzzy preferences provided in Table 1. Consequently, the results for pairwise comparisons performed in steps 3 and 4 are provided in Tables 3 and 4, respectively.

**Table 3:** Spherical fuzzy pairwise comparisons of best-to-other criteria

Criteria	Load flexibility (C_1_)	Accessibility (C_2_)	Cost (C_3_)
Best criterion (C_3_)	(0.9, 0.1, 0.1)	(0.6, 0.4, 0.4)	(0.5, 0.5, 0.5)

**Table 4:** Spherical fuzzy pairwise comparisons of other-to-worst criteria

Criteria	Worst criterion (C_1_)
Load flexibility (C_1_)	(0.5, 0.5, 0.5)
Accessibility (C_2_)	(0.7, 0.3, 0.3)
Cost (C_3_)	(0.9, 0.1, 0.1)

Table 3 is designed for the spherical fuzzy pairwise comparisons of best to all other criteria. Also, in the same way, spherical fuzzy pairwise comparisons of others-to-worst criteria are shown in Table 4,

The spherical fuzzy pairwise comparison vectors are shown based on Tables 3 and 4 as follows:

$${\widetilde{A}}_{sB}=\left(\left(0.9, 0.1, 0.1\right),\left(0.6, 0.4, 0.4\right),\left(0.5, 0.5, 0.5\right)\right)$$

$${\widetilde{A}}_{sw}=\left(\left(0.5, 0.5, 0.5\right),\left(0.7, 0.3, 0.3\right),\left(0.9, 0.1, 0.1\right)\right)$$

By considering the decision-maker's selection of the most favorable and unfavorable criteria, the Eq. (16) is formulated according to step 5 of the Sect. 3.2 in the following manner:

$$\mathrm{Min }\left({\mu }_{k}, {v}_{k}, {\pi }_{k}\right)$$

$$\mathrm{Such\,that}$$

23 $$\left\{\begin{array}{l}\Big\vert\frac{\left({\mu }_{3}^{w}, {v}_{3}^{w}, {\pi }_{3}^{w}\right)}{\left({\mu }_{1}^{w}, {v}_{1}^{w}, {\pi }_{1}^{w}\right)}-\left(0.9, 0.1, 0.1\right)\Big\vert\le \left({\mu }_{k}, {v}_{k}, {\pi }_{k}\right)\\ \Big\vert\frac{\left({\mu }_{3}^{w}, {v}_{3}^{w}, {\pi }_{3}^{w}\right)}{\left({\mu }_{2}^{w}, {v}_{2}^{w}, {\pi }_{2}^{w}\right)}-\left(0.6, 0.4, 0.4\right)\Big\vert\le \left({\mu }_{k}, {v}_{k}, {\pi }_{k}\right)\\ \Big\vert\frac{\left({\mu }_{2}^{w}, {v}_{2}^{w}, {\pi }_{2}^{w}\right)}{\left({\mu }_{1}^{w}, {v}_{1}^{w}, {\pi }_{1}^{w}\right)}-\left(0.7, 0.3, 0.3\right)\Big\vert\le \left({\mu }_{k}, {v}_{k}, {\pi }_{k}\right)\\ \sum\limits_{j=1}^{n}\left(\left({\mu }_{j}\right)*\left(1-{v}_{j}\right)*\left(1-{\pi }_{j}\right)\right)=1 \\ 0\le {\mu }_{j}+{v}_{j}\le 1 \,\mathrm{for\,all}\, j \\ 0\le {\mu }_{j}^{2}+{v}_{j}^{2}+{\pi }_{j}^{2}\le 1 \,\mathrm{for\,all}\, j \\ j=1, 2, 3\end{array}\right.$$

Considering the spherical fuzzy pairwise comparisons in Tables 3 and 4, the problem (23) is written as follows:

$$Z=\left({\mu }_{k}\right)*\left(1-{v}_{k}\right)*\left(1-{\pi }_{k}\right)$$

$$\mathrm{Such\,that}$$

$$\begin{aligned} &({\mu }_{3}^{w}, {v}_{3}^{w}, {\pi }_{3}^{w}) \le ({{({\mu }_{k}{\mu }_{1})}^{2}+{(0.9{\mu }_{1})}^{2}-({({\mu }_{k}{\mu }_{1})}^{2}*{(0.9{\mu }_{1})}^{2})}^\frac{1}{2},\\ & {({v}_{k}^{2}+{v}_{1}^{2}-{v}_{k}^{2}{v}_{1}^{2})}^\frac{1}{2}*{(0.01+{v}_{1}^{2}-0.01{v}_{1}^{2})}^\frac{1}{2}, \\ &(((1-{(0.9{\mu }_{1})}^{2})((1-{v}_{1}^{2}){\pi }_{k}^{2}+(1-{v}_{k}^{2}){\pi }_{1}^{2}-{\pi }_{k}^{2}{\pi }_{1}^{2}))\\&+((1-{({\mu }_{k}{\mu }_{1})}^{2})((1-{v}_{1}^{2})0.01+(1-0.01){\pi }_{1}^{2}-0.01{\pi }_{1}^{2}))\\ &-(((1-{v}_{1}^{2}){\pi }_{k}^{2}+(1-{v}_{k}^{2}){\pi }_{1}^{2}-{\pi }_{k}^{2}{\pi }_{1}^{2})((1-{v}_{1}^{2})0.01\\&+(1-0.01){\pi }_{1}^{2}-0.01{\pi }_{1}^{2})))^{1/2})\end{aligned}$$

$$\begin{aligned}&({\mu }_{3}^{w}, {v}_{3}^{w}, {\pi }_{3}^{w})\ge -({{({\mu }_{k}{\mu }_{1})}^{2}+{(0.9{\mu }_{1})}^{2}-({({\mu }_{k}{\mu }_{1})}^{2}*{(0.9{\mu }_{1})}^{2})}^\frac{1}{2},\\&{({v}_{k}^{2}+{v}_{1}^{2}-{v}_{k}^{2}{v}_{1}^{2})}^\frac{1}{2}*{(0.01+{v}_{1}^{2}-0.01{v}_{1}^{2})}^\frac{1}{2},\\&(((1-{(0.9{\mu }_{1})}^{2})((1-{v}_{1}^{2}){\pi }_{k}^{2}+(1-{v}_{k}^{2}){\pi }_{1}^{2}-{\pi }_{k}^{2}{\pi }_{1}^{2}))\\&+((1-{({\mu }_{k}{\mu }_{1})}^{2})((1-{v}_{1}^{2})0.01+(1-0.01){\pi }_{1}^{2}-0.01{\pi }_{1}^{2}))-(((1-{v}_{1}^{2}){\pi }_{k}^{2}\\&+(1-{v}_{k}^{2}){\pi }_{1}^{2}-{\pi }_{k}^{2}{\pi }_{1}^{2})((1-{v}_{1}^{2})0.01+(1-0.01){\pi }_{1}^{2}-0.01{\pi }_{1}^{2})))^{1/2})\end{aligned}$$

$$\begin{aligned}&({\mu }_{3}^{w}, {v}_{3}^{w}, {\pi }_{3}^{w})\le ({{({\mu }_{k}{\mu }_{2})}^{2}+{(0.6{\mu }_{2})}^{2}-({({\mu }_{k}{\mu }_{2})}^{2}*{(0.6{\mu }_{2})}^{2})}^\frac{1}{2},\\& {({v}_{k}^{2}+{v}_{2}^{2}-{v}_{k}^{2}{v}_{2}^{2})}^\frac{1}{2}*{(0.16+{v}_{2}^{2}-0.16{v}_{2}^{2})}^\frac{1}{2},\\& (((1-{(0.6{\mu }_{2})}^{2})((1-{v}_{2}^{2}){\pi }_{k}^{2}+(1-{v}_{k}^{2}){\pi }_{2}^{2}-{\pi }_{k}^{2}{\pi }_{2}^{2}))\\&+((1-{({\mu }_{k}{\mu }_{2})}^{2})((1-{v}_{2}^{2})0.16+(1-0.16){\pi }_{2}^{2}-0.16{\pi }_{2}^{2}))\\&-(((1-{v}_{2}^{2}){\pi }_{k}^{2}+(1-{v}_{k}^{2}){\pi }_{2}^{2}-{\pi }_{k}^{2}{\pi }_{2}^{2})((1-{v}_{2}^{2})0.16+(1-0.16){\pi }_{2}^{2}-0.16{\pi }_{2}^{2})))^{1/2})\end{aligned}$$

$$\begin{aligned}&({\mu }_{3}^{w}, {v}_{3}^{w}, {\pi }_{3}^{w})\ge -({{({\mu }_{k}{\mu }_{2})}^{2}+{(0.6{\mu }_{2})}^{2}-({({\mu }_{k}{\mu }_{2})}^{2}*{(0.6{\mu }_{2})}^{2})}^\frac{1}{2},\\&{({v}_{k}^{2}+{v}_{2}^{2}-{v}_{k}^{2}{v}_{2}^{2})}^\frac{1}{2}*{(0.16+{v}_{2}^{2}-0.16{v}_{2}^{2})}^\frac{1}{2},\\&(((1-{(0.6{\mu }_{2})}^{2})((1-{v}_{2}^{2}){\pi }_{k}^{2}+(1-{v}_{k}^{2}){\pi }_{2}^{2}-{\pi }_{k}^{2}{\pi }_{2}^{2}))+((1-{({\mu }_{k}{\mu }_{2})}^{2})((1-{v}_{2}^{2})0.16+(1-0.16){\pi }_{2}^{2}-0.16{\pi }_{2}^{2}))\\&-(((1-{v}_{2}^{2}){\pi }_{k}^{2}+(1-{v}_{k}^{2}){\pi }_{2}^{2}-{\pi }_{k}^{2}{\pi }_{2}^{2})((1-{v}_{2}^{2})0.16+(1-0.16){\pi }_{2}^{2}-0.16{\pi }_{2}^{2})))^{1/2})\end{aligned}$$

$$\begin{aligned}&({\mu }_{2}^{w}, {v}_{2}^{w}, {\pi }_{2}^{w})\le ({{({\mu }_{k}{\mu }_{1})}^{2}+{(0.7{\mu }_{1})}^{2}-({({\mu }_{k}{\mu }_{1})}^{2}*{(0.7{\mu }_{1})}^{2})}^\frac{1}{2},\\&{({v}_{k}^{2}+{v}_{1}^{2}-{v}_{k}^{2}{v}_{1}^{2})}^\frac{1}{2}*{(0.09+{v}_{1}^{2}-0.09{v}_{1}^{2})}^\frac{1}{2},\\&(((1-{(0.7{\mu }_{1})}^{2})((1-{v}_{1}^{2}){\pi }_{k}^{2}+(1-{v}_{k}^{2}){\pi }_{1}^{2}-{\pi }_{k}^{2}{\pi }_{1}^{2}))+((1-{({\mu }_{k}{\mu }_{1})}^{2})((1-{v}_{1}^{2})0.09+(1-0.09){\pi }_{1}^{2}-0.09{\pi }_{1}^{2}))\\&-(((1-{v}_{1}^{2}){\pi }_{k}^{2}+(1-{v}_{k}^{2}){\pi }_{1}^{2}-{\pi }_{k}^{2}{\pi }_{1}^{2})((1-{v}_{1}^{2})0.09+(1-0.09){\pi }_{1}^{2}-0.09{\pi }_{1}^{2})))^{1/2})\end{aligned}$$

$$\begin{aligned}&({\mu }_{2}^{w}, {v}_{2}^{w}, {\pi }_{2}^{w})\ge -({{({\mu }_{k}{\mu }_{1})}^{2}+{(0.7{\mu }_{1})}^{2}-({({\mu }_{k}{\mu }_{1})}^{2}*{(0.7{\mu }_{1})}^{2})}^\frac{1}{2},\\&{({v}_{k}^{2}+{v}_{1}^{2}-{v}_{k}^{2}{v}_{1}^{2})}^\frac{1}{2}*{(0.09+{v}_{1}^{2}-0.09{v}_{1}^{2})}^\frac{1}{2},\\&(((1-{(0.7{\mu }_{1})}^{2})((1-{v}_{1}^{2}){\pi }_{k}^{2}+(1-{v}_{k}^{2}){\pi }_{1}^{2}-{\pi }_{k}^{2}{\pi }_{1}^{2}))+((1-{({\mu }_{k}{\mu }_{1})}^{2})((1-{v}_{1}^{2})0.09+(1-0.09){\pi }_{1}^{2}-0.09{\pi }_{1}^{2}))\\&-(((1-{v}_{1}^{2}){\pi }_{k}^{2}+(1-{v}_{k}^{2}){\pi }_{1}^{2}-{\pi }_{k}^{2}{\pi }_{1}^{2})((1-{v}_{1}^{2})0.09+(1-0.09){\pi }_{1}^{2}-0.09{\pi }_{1}^{2})))^{1/2})\end{aligned}$$

$$\left(\left({\mu }_{1}\right)*\left(1-{v}_{1}\right)*\left(1-{\pi }_{1}\right)\right)+\left(\left({\mu }_{2}\right)*\left(1-{v}_{2}\right)*\left(1-{\pi }_{2}\right)\right)+\left(\left({\mu }_{3}\right)*\left(1-{v}_{3}\right)*\left(1-{\pi }_{3}\right)\right)=1$$

$$0\le {\mu }_{1}+{v}_{1}\le 1$$

$$0\le {\mu }_{2}+{v}_{2}\le 1$$

$$0\le {\mu }_{3}+{v}_{3}\le 1$$

$$0\le {\mu }_{1}^{2}+{v}_{1}^{2}+{\pi }_{1}^{2}\le 1$$

$$0\le {\mu }_{2}^{2}+{v}_{2}^{2}+{\pi }_{2}^{2}\le 1$$

24 $$0\le {\mu }_{3}^{2}+{v}_{3}^{2}+{\pi }_{3}^{2}\le 1$$

The spherical fuzzy weights of criteria are obtained by solving the model (24) in Lingo software. The spherical fuzzy weight of each criterion is equal to ${w}_{1}=\left(0.431, 0.312, 0.371\right)$, ${w}_{2}=\left(0.478, 0.222, 0.232\right)$, and ${w}_{3}=\left(0.581, 0.092, 0.000\right)$. The results of SFS degrees and *Z* are shown in Table 5.

**Table 5:** Results of the spherical fuzzy weight of criteria

Criteria	Spherical fuzzy weights			Crisp weights
	$\mu$	$v$	$\pi$	
Load flexibility (C_1_)	0.431	0.312	0.371	0.186
Accessibility (C_2_)	0.478	0.222	0.232	0.286
Cost (C_3_)	0.581	0.092	0.000	0.528
Z	2.239	1.715	1.069	0.111
Consistency ratio	0.0138			

According to the decision-maker’s evaluation, it is observed that the degree of membership of the best criterion (C_3_) is the highest and the worst criterion (C_1_) is the lowest, respectively. Also, the non-membership degree of the best criterion (C_3_) is the lowest and the non-membership degree of the worst criterion (C_1_) is the highest. Crisp weights are also presented in Table 5 to compare the results with Rezaei (2015) results.

Rezaei (2015) introduced the consistency ratio as an important indicator to evaluate the degree of pairwise comparisons consistency in the BWM. Comparing the criteria weights obtained using the SF-BWM with the BWM (Rezaei 2015) shows that the results were almost identical but with different accuracy. Calculating the consistency ratio ($0.111/8.04=0.0138<0.1$) based on Eq. (22) shows that the results of the SF-BWM method are very consistent compared to the traditional BWM. The spherical fuzzy preferences enable decision-makers to consider the degree of hesitancy and non-membership for pairwise comparisons to express their preferences better. Since the hesitancy degree enables the decision-makers to describe more accurate evaluations for their judgments in uncertain and ambiguous conditions. This makes the SF-BWM show better performance in vague and uncertain conditions.

### Problem II: evaluation of the waste management system

For the second problem, we considered a numerical problem by Behzad et al. (2020) where the waste management system was evaluated by using the BWM. In this problem, criteria evaluation was performed using the BWM based on real data. In the same way, the waste management problem is adopted to be solved by the SF-BWM under uncertain conditions. SF-BWM empowers us to consider the intangibility and ambiguity of decision-makers in pairwise comparisons. According to the first step mentioned in Sect. 3.2, the affective criteria should be determined. All effective criteria for evaluating the waste management system are determined by the decision-maker. In this regard, the identified criteria include waste generation (C_1_), composting waste (C_2_), Recycling waste (C_3_), Landfilling waste (C_4_), Recycling rate (C_5_), Waste to the energy rate (C_6_), and GHG emissions from waste (C_7_). In this problem, to show the application of the SF-BWM, seven criteria affecting the waste management system (Behzad et al. 2020) are considered.

According to step 2 mentioned in Sect. 3.2, the decision-maker chooses the best and worst criteria based on the mentioned points regarding the most and weakly important criteria. In this regard, the waste generation (C1) criterion is chosen as the best criterion and also the energy rate (C6) criterion is chosen as the worst criterion. After determining the effective criteria in step 1 and choosing the best and worst criteria in step 2, the pairwise comparisons are performed based on the mentioned ways in steps 3 and 4 of Sect. 3.2. In step 3, pairwise comparisons for the best criterion against all other criteria. In step 4, similar comparisons were conducted for all criteria against the worst criterion. All pairwise comparisons of steps 3 and 4 are performed using the spherical fuzzy preferences provided in Table 1. Consequently, the results for pairwise comparisons performed in steps 3 and 4 are provided in Tables 6 and 7, respectively.

**Table 6:** Spherical fuzzy pairwise comparisons of best-to-other criteria

Criteria	Waste generation (C_1_)	Composting waste (C_2_)	Recycling waste (C_3_)	Landfilling waste (C_4_)
Best criterion (C_1_)	(0.5, 0.5, 0.5)	(0.9, 0.1, 0.1)	(0.7, 0.3, 0.3)	(0.8, 0.2, 0.2)

	Recycling rate (C_5_)	Waste to the energy rate (C_6_)	GHG emissions from waste (C_7_)	
	(0.7, 0.3, 0.3)	(0.9, 0.1, 0.1)	(0.6, 0.4, 0.4)

**Table 7:** Spherical fuzzy pairwise comparisons of other-to-worst criteria

Criteria	Worst criterion (C_6_)
Waste generation (C_1_)	(0.9, 0.1, 0.1)
Composting waste (C_2_)	(0.6, 0.4, 0.4)
Recycling waste (C_3_)	(0.6, 0.4, 0.4)
Landfilling waste (C_4_)	(0.8, 0.2, 0.2)
Recycling rate (C_5_)	(0.7, 0.3, 0.3)
Waste to the energy rate (C_6_)	(0.5, 0.5, 0.5)
GHG emissions from waste (C_7_)	(0.9, 0.1, 0.1)

Table 6 is designed for the spherical fuzzy pairwise comparisons of best to all other criteria. Also, in the same way, Table 7 shows the values of spherical fuzzy pairwise comparisons of others-to-worst criteria.

The representation of the criteria vectors in the context of spherical fuzzy data is illustrated in accordance with the information provided in Tables 6 and 7, as follows.

$${\widetilde{A}}_{sB}=\left(\begin{array}{l}\left(0.5, 0.5, 0.5\right), \left(0.9, 0.1, 0.1\right),\left(0.7, 0.3, 0.3\right), \left(0.8, 0.2, 0.2\right), \left(0.7, 0.3, 0.3\right),\\ \left(0.9, 0.1, 0.1\right), \left(0.6, 0.4, 0.4\right)\end{array}\right)$$

$${\widetilde{A}}_{sw}=\left(\begin{array}{l}\left(0.9, 0.1, 0.1\right), \left(0.6, 0.4, 0.4\right), \left(0.6, 0.4, 0.4\right), \left(0.8, 0.2, 0.2\right), \left(0.7, 0.3, 0.3\right),\\ \left(0.5, 0.5, 0.5\right),\left(0.9, 0.1, 0.1\right)\end{array}\right)$$

According to the spherical fuzzy best-to-others and others-to-worst criteria vectors, problem (16) is written as follows:

$$\mathrm{Min }\left({\mu }_{k}, {v}_{k}, {\pi }_{k}\right)$$

$$\mathrm{Such that}$$

25 $$\left\{\begin{array}{l}\Big\vert\frac{\left({\mu }_{1}^{w}, {v}_{1}^{w}, {\pi }_{1}^{w}\right)}{\left({\mu }_{2}^{w}, {v}_{2}^{w}, {\pi }_{2}^{w}\right)}-\left(0.9, 0.1, 0.1\right)\Big\vert\le \left({\mu }_{k}, {v}_{k}, {\pi }_{k}\right)\\ \Big\vert\frac{\left({\mu }_{1}^{w}, {v}_{1}^{w}, {\pi }_{1}^{w}\right)}{\left({\mu }_{3}^{w}, {v}_{3}^{w}, {\pi }_{3}^{w}\right)}-\left(0.7, 0.3, 0.3\right)\Big\vert\le \left({\mu }_{k}, {v}_{k}, {\pi }_{k}\right)\\ \Big\vert\frac{\left({\mu }_{1}^{w}, {v}_{1}^{w}, {\pi }_{1}^{w}\right)}{\left({\mu }_{4}^{w}, {v}_{4}^{w}, {\pi }_{4}^{w}\right)}-\left(0.8, 0.2, 0.2\right)\Big\vert\le \left({\mu }_{k}, {v}_{k}, {\pi }_{k}\right)\\ \Big\vert\frac{\left({\mu }_{1}^{w}, {v}_{1}^{w}, {\pi }_{1}^{w}\right)}{\left({\mu }_{5}^{w}, {v}_{5}^{w}, {\pi }_{5}^{w}\right)}-\left(0.7, 0.3, 0.3\right)\Big\vert\le \left({\mu }_{k}, {v}_{k}, {\pi }_{k}\right)\\ \Big\vert\frac{\left({\mu }_{1}^{w}, {v}_{1}^{w}, {\pi }_{1}^{w}\right)}{\left({\mu }_{6}^{w}, {v}_{6}^{w}, {\pi }_{6}^{w}\right)}-\left(0.9, 0.1, 0.1\right)\Big\vert\le \left({\mu }_{k}, {v}_{k}, {\pi }_{k}\right)\\ \Big\vert\frac{\left({\mu }_{1}^{w}, {v}_{1}^{w}, {\pi }_{1}^{w}\right)}{\left({\mu }_{7}^{w}, {v}_{7}^{w}, {\pi }_{7}^{w}\right)}-\left(0.6, 0.4, 0.4\right)\Big\vert\le \left({\mu }_{k}, {v}_{k}, {\pi }_{k}\right)\\ \Big\vert\frac{\left({\mu }_{2}^{w}, {v}_{2}^{w}, {\pi }_{2}^{w}\right)}{\left({\mu }_{6}^{w}, {v}_{6}^{w}, {\pi }_{6}^{w}\right)}-\left(0.6, 0.4, 0.4\right)\Big\vert\le \left({\mu }_{k}, {v}_{k}, {\pi }_{k}\right)\\ \Big\vert\frac{\left({\mu }_{3}^{w}, {v}_{3}^{w}, {\pi }_{3}^{w}\right)}{\left({\mu }_{6}^{w}, {v}_{6}^{w}, {\pi }_{6}^{w}\right)}-\left(0.6, 0.4, 0.4\right)\Big\vert\le \left({\mu }_{k}, {v}_{k}, {\pi }_{k}\right)\\ \Big\vert\frac{\left({\mu }_{4}^{w}, {v}_{4}^{w}, {\pi }_{4}^{w}\right)}{\left({\mu }_{6}^{w}, {v}_{6}^{w}, {\pi }_{6}^{w}\right)}-\left(0.8, 0.2, 0.2\right)\Big\vert\le \left({\mu }_{k}, {v}_{k}, {\pi }_{k}\right)\\ \Big\vert\frac{\left({\mu }_{5}^{w}, {v}_{5}^{w}, {\pi }_{5}^{w}\right)}{\left({\mu }_{6}^{w}, {v}_{6}^{w}, {\pi }_{6}^{w}\right)}-\left(0.7, 0.3, 0.3\right)\Big\vert\le \left({\mu }_{k}, {v}_{k}, {\pi }_{k}\right)\\ \Big\vert\frac{\left({\mu }_{7}^{w}, {v}_{7}^{w}, {\pi }_{7}^{w}\right)}{\left({\mu }_{6}^{w}, {v}_{6}^{w}, {\pi }_{6}^{w}\right)}-\left(0.9, 0.1, 0.1\right)\Big\vert\le \left({\mu }_{k}, {v}_{k}, {\pi }_{k}\right)\\ \sum\limits_{j=1}^{n}\left(\left({\mu }_{j}\right)*\left(1-{v}_{j}\right)*\left(1-{\pi }_{j}\right)\right)=1 \\ 0\le {\mu }_{j}+{v}_{j}\le 1 \,\mathrm{for\,all}\, j \\ {\pi }_{j}=1- \left({\mu }_{j}\right)-\left({v}_{j}\right) \,\mathrm{for\,all}\, j \\ 0\le {\mu }_{j}^{2}+{v}_{j}^{2}+{\pi }_{j}^{2}\le 1 \,\mathrm{for\,all}\, j \\ j=1, 2, 3\end{array}\right.$$

Considering the spherical fuzzy pairwise comparisons in Tables 6 and 7, the problem (25) is written as follows:

$$Z=\left({\mu }_{k}\right)*\left(1-{v}_{k}\right)*\left(1-{\pi }_{k}\right)$$

Such that

$$\begin{aligned}&{(\mu }_{1}, {v}_{1},{\pi }_{1}) \le {{({\mu }_{k}{\mu }_{2})}^{2}+{(0.9{\mu }_{2})}^{2}-({({\mu }_{k}{\mu }_{2})}^{2}*{(0.9{\mu }_{2})}^{2})}^\frac{1}{2},\\&{({v}_{k}^{2}+{v}_{2}^{2}-{v}_{k}^{2}{v}_{2}^{2})}^\frac{1}{2}*{(0.01+{v}_{2}^{2}-0.01{v}_{2}^{2})}^\frac{1}{2},\\&(((1-{(0.9{\mu }_{2})}^{2})((1-{v}_{2}^{2}){\pi }_{k}^{2}+(1-{v}_{k}^{2}){\pi }_{2}^{2}-{\pi }_{k}^{2}{\pi }_{2}^{2}))+((1-{({\mu }_{k}{\mu }_{2})}^{2})((1-{v}_{2}^{2})0.01+(1-0.01){\pi }_{2}^{2}-0.01{\pi }_{2}^{2}))\\&-(((1-{v}_{2}^{2}){\pi }_{k}^{2}+(1-{v}_{k}^{2}){\pi }_{2}^{2}-{\pi }_{k}^{2}{\pi }_{2}^{2})((1-{v}_{2}^{2})0.01+(1-0.01){\pi }_{2}^{2}-0.01{\pi }_{2}^{2})))^{1/2}\end{aligned}$$

$$\begin{aligned}&{(\mu }_{1}, {v}_{1},{\pi }_{1})\ge -({{({\mu }_{k}{\mu }_{2})}^{2}+{(0.9{\mu }_{2})}^{2}-({({\mu }_{k}{\mu }_{2})}^{2}*{(0.9{\mu }_{2})}^{2})}^\frac{1}{2}),\\&-{({v}_{k}^{2}+{v}_{2}^{2}-{v}_{k}^{2}{v}_{2}^{2})}^\frac{1}{2}*{(0.01+{v}_{2}^{2}-0.01{v}_{2}^{2})}^\frac{1}{2},\\&-(((1-{(0.9{\mu }_{2})}^{2})((1-{v}_{2}^{2}){\pi }_{k}^{2}+(1-{v}_{k}^{2}){\pi }_{2}^{2}-{\pi }_{k}^{2}{\pi }_{2}^{2}))+((1-{({\mu }_{k}{\mu }_{2})}^{2})((1-{v}_{2}^{2})0.01+(1-0.01){\pi }_{2}^{2}-0.01{\pi }_{2}^{2}))\\&-(((1-{v}_{2}^{2}){\pi }_{k}^{2}+(1-{v}_{k}^{2}){\pi }_{2}^{2}-{\pi }_{k}^{2}{\pi }_{2}^{2})((1-{v}_{2}^{2})0.01+(1-0.01){\pi }_{2}^{2}-0.01{\pi }_{2}^{2})))^{1/2}\end{aligned}$$

$$\begin{aligned}&{(\mu }_{1}, {v}_{1},{\pi }_{1})\le {{({\mu }_{k}{\mu }_{3})}^{2}+{(0.7{\mu }_{3})}^{2}-({({\mu }_{k}{\mu }_{3})}^{2}*{(0.7{\mu }_{3})}^{2})}^\frac{1}{2},\\&{({v}_{k}^{2}+{v}_{3}^{2}-{v}_{k}^{2}{v}_{3}^{2})}^\frac{1}{2}*{(0.09+{v}_{3}^{2}-0.09{v}_{3}^{2})}^\frac{1}{2},\\&(((1-{(0.7{\mu }_{3})}^{2})((1-{v}_{3}^{2}){\pi }_{k}^{2}+(1-{v}_{k}^{2}){\pi }_{3}^{2}-{\pi }_{k}^{2}{\pi }_{3}^{2}))+((1-{({\mu }_{k}{\mu }_{3})}^{2})((1-{v}_{3}^{2})0.09+(1-0.09){\pi }_{3}^{2}-0.09{\pi }_{3}^{2}))\\&-(((1-{v}_{3}^{2}){\pi }_{k}^{2}+(1-{v}_{k}^{2}){\pi }_{3}^{2}-{\pi }_{k}^{2}{\pi }_{3}^{2})((1-{v}_{3}^{2})0.09+(1-0.09){\pi }_{3}^{2}-0.09{\pi }_{3}^{2})))^{1/2}\end{aligned}$$

$$\begin{aligned}&{(\mu }_{1}, {v}_{1},{\pi }_{1})\ge -({{({\mu }_{k}{\mu }_{3})}^{2}+{(0.7{\mu }_{3})}^{2}-({({\mu }_{k}{\mu }_{3})}^{2}*{(0.7{\mu }_{3})}^{2})}^\frac{1}{2}),\\&-{({v}_{k}^{2}+{v}_{3}^{2}-{v}_{k}^{2}{v}_{3}^{2})}^\frac{1}{2}*{(0.09+{v}_{3}^{2}-0.09{v}_{3}^{2})}^\frac{1}{2},\\&-(((1-{(0.7{\mu }_{3})}^{2})((1-{v}_{3}^{2}){\pi }_{k}^{2}+(1-{v}_{k}^{2}){\pi }_{3}^{2}-{\pi }_{k}^{2}{\pi }_{3}^{2}))+((1-{({\mu }_{k}{\mu }_{3})}^{2})((1-{v}_{3}^{2})0.09+(1-0.09){\pi }_{3}^{2}-0.09{\pi }_{3}^{2}))\\&-(((1-{v}_{3}^{2}){\pi }_{k}^{2}+(1-{v}_{k}^{2}){\pi }_{3}^{2}-{\pi }_{k}^{2}{\pi }_{3}^{2})((1-{v}_{3}^{2})0.09+(1-0.09){\pi }_{3}^{2}-0.09{\pi }_{3}^{2})))^{1/2}\end{aligned}$$

$$\begin{aligned}&{(\mu }_{1}, {v}_{1},{\pi }_{1})\le {{({\mu }_{k}{\mu }_{4})}^{2}+{(0.8{\mu }_{4})}^{2}-({({\mu }_{k}{\mu }_{4})}^{2}*{(0.8{\mu }_{4})}^{2})}^\frac{1}{2},\\&{({v}_{k}^{2}+{v}_{4}^{2}-{v}_{k}^{2}{v}_{4}^{2})}^\frac{1}{2}*{(0.04+{v}_{4}^{2}-0.04{v}_{4}^{2})}^\frac{1}{2},\\&(((1-{(0.8{\mu }_{4})}^{2})((1-{v}_{4}^{2}){\pi }_{k}^{2}+(1-{v}_{k}^{2}){\pi }_{4}^{2}-{\pi }_{k}^{2}{\pi }_{4}^{2}))+((1-{({\mu }_{k}{\mu }_{4})}^{2})((1-{v}_{4}^{2})0.04+(1-0.04){\pi }_{4}^{2}-0.04{\pi }_{4}^{2}))\\&-(((1-{v}_{4}^{2}){\pi }_{k}^{2}+(1-{v}_{k}^{2}){\pi }_{4}^{2}-{\pi }_{k}^{2}{\pi }_{4}^{2})((1-{v}_{4}^{2})0.04+(1-0.04){\pi }_{4}^{2}-0.04{\pi }_{4}^{2})))^{1/2}\end{aligned}$$

$$\begin{aligned}&{(\mu }_{1}, {v}_{1},{\pi }_{1})\ge -({{({\mu }_{k}{\mu }_{4})}^{2}+{(0.8{\mu }_{4})}^{2}-({({\mu }_{k}{\mu }_{4})}^{2}*{(0.8{\mu }_{4})}^{2})}^\frac{1}{2}),\\&-{({v}_{k}^{2}+{v}_{4}^{2}-{v}_{k}^{2}{v}_{4}^{2})}^\frac{1}{2}*{(0.04+{v}_{4}^{2}-0.04{v}_{4}^{2})}^\frac{1}{2},\\&-(((1-{(0.8{\mu }_{4})}^{2})((1-{v}_{4}^{2}){\pi }_{k}^{2}+(1-{v}_{k}^{2}){\pi }_{4}^{2}-{\pi }_{k}^{2}{\pi }_{4}^{2}))+((1-{({\mu }_{k}{\mu }_{4})}^{2})((1-{v}_{4}^{2})0.04+(1-0.04){\pi }_{4}^{2}-0.04{\pi }_{4}^{2}))\\&-(((1-{v}_{4}^{2}){\pi }_{k}^{2}+(1-{v}_{k}^{2}){\pi }_{4}^{2}-{\pi }_{k}^{2}{\pi }_{4}^{2})((1-{v}_{4}^{2})0.04+(1-0.04){\pi }_{4}^{2}-0.04{\pi }_{4}^{2})))^{1/2}\end{aligned}$$

$$\begin{aligned}&{(\mu }_{1}, {v}_{1},{\pi }_{1})\le {{({\mu }_{k}{\mu }_{5})}^{2}+{(0.7{\mu }_{5})}^{2}-({({\mu }_{k}{\mu }_{5})}^{2}*{(0.7{\mu }_{5})}^{2})}^\frac{1}{2},\\&{({v}_{k}^{2}+{v}_{5}^{2}-{v}_{k}^{2}{v}_{5}^{2})}^\frac{1}{2}*{(0.09+{v}_{5}^{2}-0.09{v}_{5}^{2})}^\frac{1}{2},\\&(((1-{(0.7{\mu }_{5})}^{2})((1-{v}_{5}^{2}){\pi }_{k}^{2}+(1-{v}_{k}^{2}){\pi }_{5}^{2}-{\pi }_{k}^{2}{\pi }_{5}^{2}))+((1-{({\mu }_{k}{\mu }_{5})}^{2})((1-{v}_{5}^{2})0.09+(1-0.09){\pi }_{5}^{2}-0.09{\pi }_{5}^{2}))\\&-(((1-{v}_{5}^{2}){\pi }_{k}^{2}+(1-{v}_{k}^{2}){\pi }_{5}^{2}-{\pi }_{k}^{2}{\pi }_{5}^{2})((1-{v}_{5}^{2})0.09+(1-0.09){\pi }_{5}^{2}-0.09{\pi }_{5}^{2})))^{1/2}\end{aligned}$$

$$\begin{aligned}&{(\mu }_{1}, {v}_{1},{\pi }_{1})\ge -({{({\mu }_{k}{\mu }_{5})}^{2}+{(0.7{\mu }_{5})}^{2}-({({\mu }_{k}{\mu }_{5})}^{2}*{(0.7{\mu }_{5})}^{2})}^\frac{1}{2}),\\&-{({v}_{k}^{2}+{v}_{5}^{2}-{v}_{k}^{2}{v}_{5}^{2})}^\frac{1}{2}*{(0.09+{v}_{5}^{2}-0.09{v}_{5}^{2})}^\frac{1}{2},\\&-(((1-{(0.7{\mu }_{5})}^{2})((1-{v}_{5}^{2}){\pi }_{k}^{2}+(1-{v}_{k}^{2}){\pi }_{5}^{2}-{\pi }_{k}^{2}{\pi }_{5}^{2}))+((1-{({\mu }_{k}{\mu }_{5})}^{2})((1-{v}_{5}^{2})0.09+(1-0.09){\pi }_{5}^{2}-0.09{\pi }_{5}^{2}))\\&-(((1-{v}_{5}^{2}){\pi }_{k}^{2}+(1-{v}_{k}^{2}){\pi }_{5}^{2}-{\pi }_{k}^{2}{\pi }_{5}^{2})((1-{v}_{5}^{2})0.09+(1-0.09){\pi }_{5}^{2}-0.09{\pi }_{5}^{2})))^{1/2}\end{aligned}$$

$$\begin{aligned}&{(\mu }_{1}, {v}_{1},{\pi }_{1})\le {{({\mu }_{k}{\mu }_{6})}^{2}+{(0.9{\mu }_{6})}^{2}-({({\mu }_{k}{\mu }_{6})}^{2}*{(0.9{\mu }_{6})}^{2})}^\frac{1}{2},\\&{({v}_{k}^{2}+{v}_{6}^{2}-{v}_{k}^{2}{v}_{6}^{2})}^\frac{1}{2}*{(0.01+{v}_{6}^{2}-0.01{v}_{6}^{2})}^\frac{1}{2},\\&(((1-{(0.9{\mu }_{6})}^{2})((1-{v}_{6}^{2}){\pi }_{k}^{2}+(1-{v}_{k}^{2}){\pi }_{6}^{2}-{\pi }_{k}^{2}{\pi }_{6}^{2}))+((1-{({\mu }_{k}{\mu }_{6})}^{2})((1-{v}_{6}^{2})0.01+(1-0.01){\pi }_{6}^{2}-0.01{\pi }_{6}^{2}))\\&-(((1-{v}_{6}^{2}){\pi }_{k}^{2}+(1-{v}_{k}^{2}){\pi }_{6}^{2}-{\pi }_{k}^{2}{\pi }_{6}^{2})((1-{v}_{6}^{2})0.01+(1-0.01){\pi }_{6}^{2}-0.01{\pi }_{6}^{2})))^{1/2}\end{aligned}$$

$$\begin{aligned}&{(\mu }_{1}, {v}_{1},{\pi }_{1})\ge -({{({\mu }_{k}{\mu }_{6})}^{2}+{(0.9{\mu }_{6})}^{2}-({({\mu }_{k}{\mu }_{6})}^{2}*{(0.9{\mu }_{6})}^{2})}^\frac{1}{2}),\\&-{({v}_{k}^{2}+{v}_{6}^{2}-{v}_{k}^{2}{v}_{6}^{2})}^\frac{1}{2}*{(0.01+{v}_{6}^{2}-0.01{v}_{6}^{2})}^\frac{1}{2},\\&-(((1-{(0.9{\mu }_{6})}^{2})((1-{v}_{6}^{2}){\pi }_{k}^{2}+(1-{v}_{k}^{2}){\pi }_{6}^{2}-{\pi }_{k}^{2}{\pi }_{6}^{2}))+((1-{({\mu }_{k}{\mu }_{6})}^{2})((1-{v}_{6}^{2})0.01+(1-0.01){\pi }_{6}^{2}-0.01{\pi }_{6}^{2}))\\&-(((1-{v}_{6}^{2}){\pi }_{k}^{2}+(1-{v}_{k}^{2}){\pi }_{6}^{2}-{\pi }_{k}^{2}{\pi }_{6}^{2})((1-{v}_{6}^{2})0.01+(1-0.01){\pi }_{6}^{2}-0.01{\pi }_{6}^{2})))^{1/2}\end{aligned}$$

$$\begin{aligned}&{(\mu }_{1}, {v}_{1},{\pi }_{1})\le {{({\mu }_{k}{\mu }_{7})}^{2}+{(0.6{\mu }_{7})}^{2}-({({\mu }_{k}{\mu }_{7})}^{2}*{(0.6{\mu }_{7})}^{2})}^\frac{1}{2},\\&{({v}_{k}^{2}+{v}_{7}^{2}-{v}_{k}^{2}{v}_{7}^{2})}^\frac{1}{2}*{(0.16+{v}_{7}^{2}-0.16{v}_{7}^{2})}^\frac{1}{2},\\&(((1-{(0.6{\mu }_{7})}^{2})((1-{v}_{7}^{2}){\pi }_{k}^{2}+(1-{v}_{k}^{2}){\pi }_{7}^{2}-{\pi }_{k}^{2}{\pi }_{7}^{2}))+((1-{({\mu }_{k}{\mu }_{7})}^{2})((1-{v}_{7}^{2})0.16+(1-0.16){\pi }_{7}^{2}-0.16{\pi }_{7}^{2}))\\&-(((1-{v}_{7}^{2}){\pi }_{k}^{2}+(1-{v}_{k}^{2}){\pi }_{7}^{2}-{\pi }_{k}^{2}{\pi }_{7}^{2})((1-{v}_{7}^{2})0.16+(1-0.16){\pi }_{7}^{2}-0.16{\pi }_{7}^{2})))^{1/2}\end{aligned}$$

$$\begin{aligned}{(\mu }_{1}, {v}_{1},{\pi }_{1})\ge -({{({\mu }_{k}{\mu }_{7})}^{2}+{(0.6{\mu }_{7})}^{2}-({({\mu }_{k}{\mu }_{7})}^{2}*{(0.6{\mu }_{7})}^{2})}^\frac{1}{2}),-{({v}_{k}^{2}+{v}_{7}^{2}-{v}_{k}^{2}{v}_{7}^{2})}^\frac{1}{2}*{(0.16+{v}_{7}^{2}-0.16{v}_{7}^{2})}^\frac{1}{2},&\\-(((1-{(0.6{\mu }_{7})}^{2})((1-{v}_{7}^{2}){\pi }_{k}^{2}+(1-{v}_{k}^{2}){\pi }_{7}^{2}-{\pi }_{k}^{2}{\pi }_{7}^{2}))+((1-({\mu }_{k}{\mu }_{7})^{2})((1-{v}_{7}^{2})0.16+(1-0.16){\pi }_{7}^{2}-0.16{\pi }_{7}^{2}))&\\-(((1-{v}_{7}^{2}){\pi }_{k}^{2}+(1-{v}_{k}^{2}){\pi }_{7}^{2}-{\pi }_{k}^{2}{\pi }_{7}^{2})((1-{v}_{7}^{2})0.16+(1-0.16){\pi }_{7}^{2}-0.16{\pi }_{7}^{2})))^{1/2}\end{aligned}$$

$$\begin{aligned}&{(\mu }_{2}, {v}_{2},{\pi }_{2})\le {{({\mu }_{k}{\mu }_{6})}^{2}+{(0.6{\mu }_{6})}^{2}-({({\mu }_{k}{\mu }_{6})}^{2}*{(0.6{\mu }_{6})}^{2})}^\frac{1}{2},\\&{({v}_{k}^{2}+{v}_{6}^{2}-{v}_{k}^{2}{v}_{6}^{2})}^\frac{1}{2}*{(0.16+{v}_{6}^{2}-0.16{v}_{6}^{2})}^\frac{1}{2},\\&(((1-{(0.6{\mu }_{6})}^{2})((1-{v}_{6}^{2}){\pi }_{k}^{2}+(1-{v}_{k}^{2}){\pi }_{6}^{2}-{\pi }_{k}^{2}{\pi }_{6}^{2}))+((1-{({\mu }_{k}{\mu }_{6})}^{2})((1-{v}_{6}^{2})0.16+(1-0.16){\pi }_{6}^{2}-0.16{\pi }_{6}^{2}))\\&-(((1-{v}_{6}^{2}){\pi }_{k}^{2}+(1-{v}_{k}^{2}){\pi }_{6}^{2}-{\pi }_{k}^{2}{\pi }_{6}^{2})((1-{v}_{6}^{2})0.16+(1-0.16){\pi }_{6}^{2}-0.16{\pi }_{6}^{2})))^{1/2}\end{aligned}$$

$$\begin{aligned}&{(\mu }_{2}, {v}_{2},{\pi }_{2})\ge -({{({\mu }_{k}{\mu }_{6})}^{2}+{(0.6{\mu }_{6})}^{2}-({({\mu }_{k}{\mu }_{6})}^{2}*{(0.6{\mu }_{6})}^{2})}^\frac{1}{2}),\\&-{({v}_{k}^{2}+{v}_{6}^{2}-{v}_{k}^{2}{v}_{6}^{2})}^\frac{1}{2}*{(0.16+{v}_{6}^{2}-0.16{v}_{6}^{2})}^\frac{1}{2},\\&-(((1-{(0.6{\mu }_{6})}^{2})((1-{v}_{6}^{2}){\pi }_{k}^{2}+(1-{v}_{k}^{2}){\pi }_{6}^{2}-{\pi }_{k}^{2}{\pi }_{6}^{2}))+((1-{({\mu }_{k}{\mu }_{6})}^{2})((1-{v}_{6}^{2})0.16+(1-0.16){\pi }_{6}^{2}-0.16{\pi }_{6}^{2}))\\&-(((1-{v}_{6}^{2}){\pi }_{k}^{2}+(1-{v}_{k}^{2}){\pi }_{6}^{2}-{\pi }_{k}^{2}{\pi }_{6}^{2})((1-{v}_{6}^{2})0.16+(1-0.16){\pi }_{6}^{2}-0.16{\pi }_{6}^{2})))^{1/2}\end{aligned}$$

$$\begin{aligned}&{(\mu }_{3}, {v}_{3},{\pi }_{3})\le {{({\mu }_{k}{\mu }_{6})}^{2}+{(0.6{\mu }_{6})}^{2}-({({\mu }_{k}{\mu }_{6})}^{2}*{(0.6{\mu }_{6})}^{2})}^\frac{1}{2},\\&{({v}_{k}^{2}+{v}_{6}^{2}-{v}_{k}^{2}{v}_{6}^{2})}^\frac{1}{2}*{(0.16+{v}_{6}^{2}-0.16{v}_{6}^{2})}^\frac{1}{2},\\&(((1-{(0.6{\mu }_{6})}^{2})((1-{v}_{6}^{2}){\pi }_{k}^{2}+(1-{v}_{k}^{2}){\pi }_{6}^{2}-{\pi }_{k}^{2}{\pi }_{6}^{2}))+((1-{({\mu }_{k}{\mu }_{6})}^{2})((1-{v}_{6}^{2})0.16+(1-0.16){\pi }_{6}^{2}-0.16{\pi }_{6}^{2}))\\&-(((1-{v}_{6}^{2}){\pi }_{k}^{2}+(1-{v}_{k}^{2}){\pi }_{6}^{2}-{\pi }_{k}^{2}{\pi }_{6}^{2})((1-{v}_{6}^{2})0.16+(1-0.16){\pi }_{6}^{2}-0.16{\pi }_{6}^{2})))^{1/2}\end{aligned}$$

$$\begin{aligned}&{(\mu }_{3}, {v}_{3},{\pi }_{3})\ge -({{({\mu }_{k}{\mu }_{6})}^{2}+{(0.6{\mu }_{6})}^{2}-({({\mu }_{k}{\mu }_{6})}^{2}*{(0.6{\mu }_{6})}^{2})}^\frac{1}{2}),\\&-{({v}_{k}^{2}+{v}_{6}^{2}-{v}_{k}^{2}{v}_{6}^{2})}^\frac{1}{2}*{(0.16+{v}_{6}^{2}-0.16{v}_{6}^{2})}^\frac{1}{2}, \\&-(((1-{(0.6{\mu }_{6})}^{2})((1-{v}_{6}^{2}){\pi }_{k}^{2}+(1-{v}_{k}^{2}){\pi }_{6}^{2}-{\pi }_{k}^{2}{\pi }_{6}^{2}))+((1-{({\mu }_{k}{\mu }_{6})}^{2})((1-{v}_{6}^{2})0.16+(1-0.16){\pi }_{6}^{2}-0.16{\pi }_{6}^{2}))\\&-(((1-{v}_{6}^{2}){\pi }_{k}^{2}+(1-{v}_{k}^{2}){\pi }_{6}^{2}-{\pi }_{k}^{2}{\pi }_{6}^{2})((1-{v}_{6}^{2})0.16+(1-0.16){\pi }_{6}^{2}-0.16{\pi }_{6}^{2})))^{1/2}\end{aligned}$$

$$\begin{aligned}&{(\mu }_{4}, {v}_{4},{\pi }_{4})\le {{({\mu }_{k}{\mu }_{6})}^{2}+{(0.8{\mu }_{6})}^{2}-({({\mu }_{k}{\mu }_{6})}^{2}*{(0.8{\mu }_{6})}^{2})}^\frac{1}{2},\\&{({v}_{k}^{2}+{v}_{6}^{2}-{v}_{k}^{2}{v}_{6}^{2})}^\frac{1}{2}*{(0.04+{v}_{6}^{2}-0.04{v}_{6}^{2})}^\frac{1}{2},\\&(((1-{(0.8{\mu }_{6})}^{2})((1-{v}_{6}^{2}){\pi }_{k}^{2}+(1-{v}_{k}^{2}){\pi }_{6}^{2}-{\pi }_{k}^{2}{\pi }_{6}^{2}))+((1-{({\mu }_{k}{\mu }_{6})}^{2})((1-{v}_{6}^{2})0.04+(1-0.04){\pi }_{6}^{2}-0.04{\pi }_{6}^{2}))\\&-(((1-{v}_{6}^{2}){\pi }_{k}^{2}+(1-{v}_{k}^{2}){\pi }_{6}^{2}-{\pi }_{k}^{2}{\pi }_{6}^{2})((1-{v}_{6}^{2})0.04+(1-0.04){\pi }_{6}^{2}-0.04{\pi }_{6}^{2})))^{1/2}\end{aligned}$$

$$\begin{aligned}&{(\mu }_{4}, {v}_{4},{\pi }_{4})\ge -({{({\mu }_{k}{\mu }_{6})}^{2}+{(0.8{\mu }_{6})}^{2}-({({\mu }_{k}{\mu }_{6})}^{2}*{(0.8{\mu }_{6})}^{2})}^\frac{1}{2}),\\&-{({v}_{k}^{2}+{v}_{6}^{2}-{v}_{k}^{2}{v}_{6}^{2})}^\frac{1}{2}*{(0.04+{v}_{6}^{2}-0.04{v}_{6}^{2})}^\frac{1}{2},\\&-(((1-{(0.8{\mu }_{6})}^{2})((1-{v}_{6}^{2}){\pi }_{k}^{2}+(1-{v}_{k}^{2}){\pi }_{6}^{2}-{\pi }_{k}^{2}{\pi }_{6}^{2}))+((1-{({\mu }_{k}{\mu }_{6})}^{2})((1-{v}_{6}^{2})0.04+(1-0.04){\pi }_{6}^{2}-0.04{\pi }_{6}^{2}))\\&-(((1-{v}_{6}^{2}){\pi }_{k}^{2}+(1-{v}_{k}^{2}){\pi }_{6}^{2}-{\pi }_{k}^{2}{\pi }_{6}^{2})((1-{v}_{6}^{2})0.04+(1-0.04){\pi }_{6}^{2}-0.04{\pi }_{6}^{2})))^{1/2}\end{aligned}$$

$$\begin{aligned}&{(\mu }_{5}, {v}_{5},{\pi }_{5})\le {{({\mu }_{k}{\mu }_{6})}^{2}+{(0.7{\mu }_{6})}^{2}-({({\mu }_{k}{\mu }_{6})}^{2}*{(0.7{\mu }_{6})}^{2})}^\frac{1}{2},\\&{({v}_{k}^{2}+{v}_{6}^{2}-{v}_{k}^{2}{v}_{6}^{2})}^\frac{1}{2}*{(0.09+{v}_{6}^{2}-0.09{v}_{6}^{2})}^\frac{1}{2},\\&(((1-{(0.7{\mu }_{6})}^{2})((1-{v}_{6}^{2}){\pi }_{k}^{2}+(1-{v}_{k}^{2}){\pi }_{6}^{2}-{\pi }_{k}^{2}{\pi }_{6}^{2}))+((1-{({\mu }_{k}{\mu }_{6})}^{2})((1-{v}_{6}^{2})0.09+(1-0.09){\pi }_{6}^{2}-0.09{\pi }_{6}^{2}))\\&-(((1-{v}_{6}^{2}){\pi }_{k}^{2}+(1-{v}_{k}^{2}){\pi }_{6}^{2}-{\pi }_{k}^{2}{\pi }_{6}^{2})((1-{v}_{6}^{2})0.09+(1-0.09){\pi }_{6}^{2}-0.09{\pi }_{6}^{2})))^{1/2}\end{aligned}$$

$$\begin{aligned}&{(\mu }_{5}, {v}_{5},{\pi }_{5})\ge -({{({\mu }_{k}{\mu }_{6})}^{2}+{(0.7{\mu }_{6})}^{2}-({({\mu }_{k}{\mu }_{6})}^{2}*{(0.7{\mu }_{6})}^{2})}^\frac{1}{2}),\\&-{({v}_{k}^{2}+{v}_{6}^{2}-{v}_{k}^{2}{v}_{6}^{2})}^\frac{1}{2}*{(0.09+{v}_{6}^{2}-0.09{v}_{6}^{2})}^\frac{1}{2},\\&-(((1-{(0.7{\mu }_{6})}^{2})((1-{v}_{6}^{2}){\pi }_{k}^{2}+(1-{v}_{k}^{2}){\pi }_{6}^{2}-{\pi }_{k}^{2}{\pi }_{6}^{2}))+((1-{({\mu }_{k}{\mu }_{6})}^{2})((1-{v}_{6}^{2})0.09+(1-0.09){\pi }_{6}^{2}-0.09{\pi }_{6}^{2}))\\&-(((1-{v}_{6}^{2}){\pi }_{k}^{2}+(1-{v}_{k}^{2}){\pi }_{6}^{2}-{\pi }_{k}^{2}{\pi }_{6}^{2})((1-{v}_{6}^{2})0.09+(1-0.09){\pi }_{6}^{2}-0.09{\pi }_{6}^{2})))^{1/2}\end{aligned}$$

$$\begin{aligned}&{(\mu }_{7}, {v}_{7},{\pi }_{7})\le {{({\mu }_{k}{\mu }_{6})}^{2}+{(0.9{\mu }_{6})}^{2}-({({\mu }_{k}{\mu }_{6})}^{2}*{(0.9{\mu }_{6})}^{2})}^\frac{1}{2},\\&{({v}_{k}^{2}+{v}_{6}^{2}-{v}_{k}^{2}{v}_{6}^{2})}^\frac{1}{2}*{(0.01+{v}_{6}^{2}-0.01{v}_{6}^{2})}^\frac{1}{2},\\&(((1-{(0.9{\mu }_{6})}^{2})((1-{v}_{6}^{2}){\pi }_{k}^{2}+(1-{v}_{k}^{2}){\pi }_{6}^{2}-{\pi }_{k}^{2}{\pi }_{6}^{2}))+((1-{({\mu }_{k}{\mu }_{6})}^{2})((1-{v}_{6}^{2})0.01+(1-0.01){\pi }_{6}^{2}-0.01{\pi }_{6}^{2}))\\&-(((1-{v}_{6}^{2}){\pi }_{k}^{2}+(1-{v}_{k}^{2}){\pi }_{6}^{2}-{\pi }_{k}^{2}{\pi }_{6}^{2})((1-{v}_{6}^{2})0.01+(1-0.01){\pi }_{6}^{2}-0.01{\pi }_{6}^{2})))^{1/2}\end{aligned}$$

$$\begin{aligned}&{(\mu }_{7}, {v}_{7},{\pi }_{7})\ge -({{({\mu }_{k}{\mu }_{6})}^{2}+{(0.9{\mu }_{6})}^{2}-({({\mu }_{k}{\mu }_{6})}^{2}*{(0.9{\mu }_{6})}^{2})}^\frac{1}{2}),\\&-{({v}_{k}^{2}+{v}_{6}^{2}-{v}_{k}^{2}{v}_{6}^{2})}^\frac{1}{2}*{(0.01+{v}_{6}^{2}-0.01{v}_{6}^{2})}^\frac{1}{2},\\&-(((1-{(0.9{\mu }_{6})}^{2})((1-{v}_{6}^{2}){\pi }_{k}^{2}+(1-{v}_{k}^{2}){\pi }_{6}^{2}-{\pi }_{k}^{2}{\pi }_{6}^{2}))+((1-{({\mu }_{k}{\mu }_{6})}^{2})((1-{v}_{6}^{2})0.01+(1-0.01){\pi }_{6}^{2}-0.01{\pi }_{6}^{2}))\\&-(((1-{v}_{6}^{2}){\pi }_{k}^{2}+(1-{v}_{k}^{2}){\pi }_{6}^{2}-{\pi }_{k}^{2}{\pi }_{6}^{2})((1-{v}_{6}^{2})0.01+(1-0.01){\pi }_{6}^{2}-0.01{\pi }_{6}^{2})))^{1/2}\end{aligned}$$

$$\begin{aligned}&\left(\left({\mu }_{1}\right)*\left(1-{v}_{1}\right)*\left(1-{\pi }_{1}\right)\right)+\left(\left({\mu }_{2}\right)*\left(1-{v}_{2}\right)*\left(1-{\pi }_{2}\right)\right)+\left(\left({\mu }_{3}\right)*\left(1-{v}_{3}\right)*\left(1-{\pi }_{3}\right)\right)\\&+\left(\left({\mu }_{4}\right)*\left(1-{v}_{4}\right)*\left(1-{\pi }_{4}\right)\right)+\left(\left({\mu }_{5}\right)*\left(1-{v}_{5}\right)*\left(1-{\pi }_{5}\right)\right)\\&+\left(\left({\mu }_{6}\right)*\left(1-{v}_{6}\right)*\left(1-{\pi }_{6}\right)\right)+\left(\left({\mu }_{7}\right)*\left(1-{v}_{7}\right)*\left(1-{\pi }_{7}\right)\right)=1\end{aligned}$$

$$0\le {\mu }_{1}+{v}_{1}\le 1$$

$$0\le {\mu }_{2}+{v}_{2}\le 1$$

$$0\le {\mu }_{3}+{v}_{3}\le 1$$

$$0\le {\mu }_{4}+{v}_{4}\le 1$$

$$0\le {\mu }_{5}+{v}_{5}\le 1$$

$$0\le {\mu }_{6}+{v}_{6}\le 1$$

$$0\le {\mu }_{7}+{v}_{7}\le 1$$

$$0\le {\mu }_{1}^{2}+{v}_{1}^{2}+{\pi }_{1}^{2}\le 1$$

$$0\le {\mu }_{2}^{2}+{v}_{2}^{2}+{\pi }_{2}^{2}\le 1$$

$$0\le {\mu }_{3}^{2}+{v}_{3}^{2}+{\pi }_{3}^{2}\le 1$$

$$0\le {\mu }_{4}^{2}+{v}_{4}^{2}+{\pi }_{4}^{2}\le 1$$

$$0\le {\mu }_{5}^{2}+{v}_{5}^{2}+{\pi }_{5}^{2}\le 1$$

$$0\le {\mu }_{6}^{2}+{v}_{6}^{2}+{\pi }_{6}^{2}\le 1$$

26 $$0\le {\mu }_{7}^{2}+{v}_{7}^{2}+{\pi }_{7}^{2}\le 1$$

The final weights are calculated based on the model (26). Results of spherical fuzzy criteria weights are.

$${{\text{w}}}_{1}=\left(0.186, 0.000, 0.000\right)$$,$${{\text{w}}}_{2}=\left(0.158, 0.000, 0.000\right),$$ $${{\text{w}}}_{3}=\left(0.178, 0.014, 0.141\right),$$

$${{\text{w}}}_{4}=\left(0.159, 0.006, 0.237\right),$$ $${{\text{w}}}_{5}=\left(0.180, 0.009, 0.323\right),$$ $${{\text{w}}}_{6}=\left(0.158, 0.241, 0.088\right)$$,

$${{\text{w}}}_{7}=\left(0.161, 0.005, 0.032\right)$$

The results of hesitancy, non-membership and membership degrees for all criteria and Z are shown in Table 8.

**Table 8:** Results of the spherical fuzzy weight of criteria

Criteria	Spherical fuzzy weights			Crisp weights
	$\upmu$	${\text{v}}$	$\uppi$	
Waste generation (C_1_)	0.186	0.000	0.000	0.186
Composting waste (C_2_)	0.158	0.000	0.000	0.158
Recycling waste (C_3_)	0.178	0.014	0.141	0.151
Landfilling waste (C_4_)	0.159	0.006	0.237	0.120
Recycling rate (C_5_)	0.180	0.009	0.323	0.121
Waste to the energy rate (C_6_)	0.158	0.241	0.088	0.109
GHG emissions from waste (C_7_)	0.161	0.005	0.032	0.155
*Z*	0.372	0.474	0.000	0.196
Consistency ratio	0.024			

According to the decision-maker’s opinion, it is observed that the degree of membership of the best criterion (C_1_) is the highest and the worst criterion (C_6_) is the lowest, respectively. Also, the non-membership degree of the best criterion (C_1_) is the lowest, but the non-membership degree of the worst criterion (C_6_) is not the highest, because the hesitancy degree of the worst criterion (C_6_) is the highest.

Crisp weights are also presented in Table 8 to compare the results with Behzad et al. (2020). The value of *Z* is 0.196 and ${\widetilde{a}}_{BW}$ the consistency index value is 8.04 (based on Table 2). Therefore, the consistency ratio is 0.024 and acceptable for this problem.

## Comparative analysis

This section shows using the SF-BWM method to obtain the criteria weight leads to a better consistency ratio of results. The main reason for the improvement in the consistency ratio is the consideration of decision-maker ambiguity and increasing the domain of preferences that decision-makers can use. As mentioned before, the values of the *Z* (ξ) and consistency ratio are important in showing the approvable of the weights results. The closer the consistency ratio value is to zero, the more consistent the results. In this context, a method that yields a high consistency ratio holds significant importance. Given that, in most research, the weights obtained in the subsequent step are used to calculate the ranking of options, even minor inconsistencies in the weight results can result in significant differences in the ranking of options.

The numerical problem I presented in this paper has already been solved by Rezaei (2015) using BWM. Also, Guo and Zhao (2017) have solved this problem using the fuzzy BWM. The obtained weights and consistency ratio results for three BWM, fuzzy BWM, and SF-BWM methods for this problem are shown in Table 9. The results obtained for the criteria weights show that the priority of the criteria is the same in all three methods. However, there are differences in the criteria weights values based on the proposed method that indicate the accuracy and reliability of the obtained results. The main advantage of using SF-BWM is providing a better consistency ratio. As shown in Table 9, the consistency ratio obtained for SF-BWM is threefold better than the BWM and fuzzy BWM methods.

**Table 9:** Comparison results between BWM, fuzzy BWM and SF-BWM

Criteria	Problem I: Selection of transportation mode			Problem II: Waste management system	
	BWM (Rezaei 2015)	Fuzzy BWM (Guo and Zhao 2017)	SF-BWM	BWM (Behzad et al. 2020)	SF-BWM
C_1_	0.0714	0.143	0.186	0.262	0.186
C_2_	0.3387	0.349	0.285	0.085	0.158
C_3_	0.5899	0.507	0.527	0.142	0.151
C_4_	–	–	–	0.106	0.120
C_5_	–	–	–	0.142	0.121
C_6_	–	–	–	0.050	0.109
C_7_	–	–	–	0.213	0.155
Z (ξ)	0.26	0.449	0.1111	0.163	0.196
Consistency ratio	0.0582	0.055	0.0138	0.071	0.024

Additionally, in the numerical problem II, comparing the results calculated using the SF-BWM, with the results of Behzad et al. (2020) shows that again SF-BWM has resulted in a better consistency ratio than the BWM. Consistency in the results of the weights leads to more confidence in the decisions. The comprised results of the BWM, fuzzy BWM, and SF-BWM consistency in the numerical problems show that the SF-BWM has better threefold almost strengths in this field. Also, similar to Problem I, the consistency ratio obtained for SF-BWM in Problem II is threefold better than the calculated BWM by Behzad et al. (2020).

## Conclusion and future study

The BWM proves to be a robust MCDM technique used for ascertaining the weight coefficients of decision criteria in intricate MCDM scenarios. Functioning as a vector-centric approach, the BWM utilizes pairwise comparisons to calculate the weight coefficients for decision criteria. Nevertheless, the primary benefit of the BWM, distinguishing it from other methods like AHP, lies in its structure requiring fewer pairwise comparisons. The intangibility and vagueness of some problems prevent the applying the BWM in a real-life context. In this regard, fuzzy BWM was developed to handle uncertainty in decision-makers’ opinions through triangular fuzzy sets. However, with an increase of complexity and uncertainty of MCDM problems, triangular fuzzy sets are no longer a reliable uncertain environment to decide. This stems from the recently advanced extensions of fuzzy sets developed to improve decision-makers’ preference domain. Therefore, this paper proposes the SF-BWM to address MCDM problems under uncertain and ambiguous circumstances. The SFS uses three degrees hesitancy, non-membership, and membership functions. In the SFS, hesitancy increases the decision-maker’s preference domain to express their judgments with higher reliability. Therefore, the SF-BWM provides a strong structure for decision-makers to reflect their hesitancy in uncertain conditions.

To show the applicability of SF-BWM and its efficiency compared to BWM and fuzzy BWM, two numerical problems from the literature are investigated. First, the SF-BWM is utilized to solve the supplier selection problem in Rezaei (2015). The results of the criteria weight show the order of priority of the criteria by SF-BWM is the same as Rezaei (2015). However, the criteria weight obtained by the SF-BWM is entirely consistent due to the higher consistency ratio of SF-BWM compared to BWM and fuzzy BWM. Then, in the second problem, the performance of the waste management system studied by Behzad et al. (2020) is utilized to illustrate the application of the SF-BWM. This problem’s analyzed results indicate that decision-makers in the SF-BWM express their hesitancy for judgment of pairwise comparison value leads to more accurate results. Similar to the first problem, SF-BWM obtains a better consistency ratio than others.

Regarding the limitations of the proposed method, the SF-BWM might become impractical when dealing with a large number of alternatives or criteria, as pairwise comparisons can become time-consuming and complex. Also, introducing SFS and using it to extend the BWM may increase the complexity of the decision-making process, potentially requiring more computational resources. This could be a limitation, especially for large-scale decision problems. In this regard, SFS linguistic variables involve subjective input from decision-makers. The use of SFS to extend BWM might still be subject to challenges related to obtaining consistent and reliable judgments from experts. Also, limitations could arise if there's a lack of consensus among experts.

Regarding the advantages of the proposed method to apply in solving different scientific decision problems, the better consistency results with the SF-BWM contribute to its widespread use. In conclusion, the appropriateness of using the SF-BWM in different scientific fields for decision-making problems lies in its ability to provide a clear, comparative analysis of alternatives based on decision-makers' preferences. While it has its limitations, the method's advantages make it a valuable tool when applied judiciously in contexts where its characteristics align with the nature of the decision problem and the preferences of decision-makers.

For future studies, a new approach of the SF-BWM based on groups of decision-makers can be introduced for problems with a lot of the decision-makers. As SF-BWM is a novel approach, it can be used to address real-life problems in different fields such as energy planning, waste management, supply chain management, healthcare management, transportation planning, scenario evaluation, sustainability, and circular economy. One may develop an integrated MCDM model by combining the SF-BWM with other MCDM methods such the VIKOR, TOPSIS, ELECTRE, etc. An integrated weighting model can be developed based on SF-BWM with an objective weighting method such as Shannon’s entropy to consider both subjective and objective perspectives.

## Data Availability

The datasets used and/or analyzed during the current study are available from the corresponding author on reasonable request.
